# Pairwise combinations of chemical compounds that delay yeast chronological aging through different signaling pathways display synergistic effects on the extent of aging delay

**DOI:** 10.18632/oncotarget.26553

**Published:** 2019-01-08

**Authors:** Pamela Dakik, Mélissa McAuley, Marisa Chancharoen, Darya Mitrofanova, Monica Enith Lozano Rodriguez, Jennifer Anne Baratang Junio, Vicky Lutchman, Berly Cortes, Éric Simard, Vladimir I. Titorenko

**Affiliations:** ^1^ Department of Biology, Concordia University, Montreal, Quebec, Canada; ^2^ Idunn Technologies Inc., Rosemere, Quebec, Canada

**Keywords:** yeast, cellular aging, geroprotectors, cellular signaling, gerotarget

## Abstract

We have recently discovered six plant extracts that delay yeast chronological aging. Most of them affect different nodes, edges and modules of an evolutionarily conserved network of longevity regulation that integrates certain signaling pathways and protein kinases; this network is also under control of such aging-delaying chemical compounds as spermidine and resveratrol. We have previously shown that, if a strain carrying an aging-delaying single-gene mutation affecting a certain node, edge or module of the network is exposed to some of the six plant extracts, the mutation and the plant extract enhance aging-delaying efficiencies of each other so that their combination has a synergistic effect on the extent of aging delay. We therefore hypothesized that a pairwise combination of two aging-delaying plant extracts or a combination of one of these plant extracts and spermidine or resveratrol may have a synergistic effect on the extent of aging delay only if each component of this combination targets a different element of the network. To test our hypothesis, we assessed longevity-extending efficiencies of all possible pairwise combinations of the six plant extracts or of one of them and spermidine or resveratrol in chronologically aging yeast. In support of our hypothesis, we show that only pairwise combinations of naturally-occurring chemical compounds that slow aging through different nodes, edges and modules of the network delay aging in a synergistic manner.

## INTRODUCTION

Different patient-customized combinations of Chinese plants have been for centuries used in traditional Chinese herbal medicine to prevent and treat a wide range of human diseases [1–4]. The recent use of high-throughput ″omics″, system biological and bioinformatic approaches in animal models of many human diseases and in cultured human cells has revealed that these combinations of Chinese plants affect multiple cellular and organismal processes [4, 5–8]. The term ″network pharmacology″ (also known as ″Traditional Chinese Medicine [TCM] network pharmacology″) has been introduced to describe such systemic effects of various combinations of Chinese herbs on the cellular and organismal networks integrating multiple disease-related processes [9–19]. The key tenet of the TCM network pharmacology concept is that the high therapeutic effectiveness, multitude of targeted pathologies and limited side effects of Chinese herbal medicine are due to the ability of cocktails of plant chemical compounds to affect multiple molecular targets through numerous low-affinity interactions [4, 9–22]. A body of evidence supports the notion that, by modulating different nodes, edges and modules of these disease-specific networks, a combined action of Chinese herb mixtures allows to restore cellular and organismal homeostasis differently altered in diverse human diseases [4, 8, 15, 19–22].

The two important conceptual advances in our understanding of the biology of human diseases are the following: 1) many (in not all) human diseases are due to various perturbations in complex networks that assimilate specific signaling, metabolic and other molecular pathways; and 2) a rational design of certain combinations of pure drugs and/or natural chemical compounds, each targeting different nodes, edges or modules of such networks, may yield potent multicomponent therapies with limited side effects and lowered potential of drug resistance development [23–36]. A development of such multicomponent therapies with the help of high-throughput screening methods for identifying effective combinations of therapeutic compounds represents an essential strategy of modern medicine [23–37]. This strategy has been successfully used for the development of multicomponent and multitargeted therapeutics for the treatment of complex human diseases, including cancer, infectious diseases, central nervous system disorders, Alzheimer’s disease, hypertension, chronic obstructive pulmonary disease, asthma and acquired immunodeficiency syndrome [23, 32, 34, 35, 37–49]. The significant progress in developing multicomponent and multitargeted therapeutics has been facilitated by the advances in mathematical, computational and pharmacological approaches to study compound combination effects [37, 49–58].

Incidence rates of many human chronic diseases increase with age [59–63]. Because an age-related dysregulation of certain cellular and organismal processes is the primary cause of these chronic diseases, they are considered as diseases associated with aging [60, 61, 64–66]. Among these aging-associated human diseases are cardiovascular diseases, chronic obstructive pulmonary disease, chronic kidney disease, diabetes, osteoarthritis, osteoporosis, sarcopenia, stroke, neurodegenerative diseases (including Parkinson’s, Alzheimer’s and Huntington’s diseases), and many forms of cancer [61–70]. The major aspects and basic mechanisms of aging and aging-associated pathology have been conserved over the course of evolution; they include the following: 1) the hallmark events of aging, such as the age-related accumulation of genomic damage, deterioration of telomeres, epigenetic perturbations, impairment of cellular proteostasis, deregulation of nutrient-sensing systems, decline in mitochondrial functionality, accumulation of senescent cells, decrease of the abundance and functionality of stem cells, anddeterioration of intercellular communications [68]; 2) the nutrient- and energy-sensing signaling network of longevity regulation, which integrates the insulin/insulin-like growth factor 1 (IGF-1) pathway, AMP-activated protein kinase/target of rapamycin (AMPK/TOR) pathway, cAMP/protein kinase A (cAMP/PKA) pathway and sirtuin-governed protein deacetylation module [66–68, 71–73].

Some of the known hallmarks of aging and signaling pathways of longevity regulation are specifically targeted by certain individually added chemical compounds (either synthetic drugs or natural chemicals) that delay the onset and decelerate the progression of the aging process in eukaryotes across phyla [60, 64–70, 74–76]. It needs to be emphasized that none of these individually added aging-delaying chemical compounds can affect all hallmarks of aging or modulate all signaling pathways of longevity regulation [64–67, 75, 76]. It has been therefore proposed that, if two or more aging-delaying chemical compounds each targeting a different hallmark and/or signaling pathway of aging are added together, their combination may have an additive or a synergistic effect on the extent of aging delay and longevity extension [74–77]. The following multicomponent combinations of chemical compounds have been proposed for such therapeutic multiplexing of aging delay and longevity extension: 1) a three-component mixture of epigallocatechin gallate (an activator of cAMP synthesis), N-acetyl-L-cysteine (an inhibitor of cell proliferation pathways) and myricetin (an activator of integrin signaling, DNA repair, cAMP synthesis and hypoxia signaling) [74]; 2) a seven-component mixture of rapalogs (including rapamycin and its synthetic drug analogs, all of which are inhibitors of the pro-aging TOR pathway), metformin (an activator of AMPK, which is a major cellular regulator of glucose and lipid metabolism), losartan or lisinopril (both of which are inhibitors of angiotensin II signaling), a statin (such as atorvastatin, simvastatin or lovastatin, all of which decrease blood cholesterol levels), propranolol (a non-cardioselective beta-adrenergic antagonist), aspirin (an inhibitor of cyclooxygenase) and a phosphodiesterase 5 inhibitor, in combination with physical exercise and caloric restriction (CR) diet or intermittent fasting [76, 78]; and 3) a three-component mixture of rapamycin, acarbose (an a-glucosidase inhibitor) and a cardiolipin-binding peptide [77].

Recent studies in mice have supported the notion that a combination of the aging-delaying chemical compounds that target different aging-associated processes may exhibit a synergistic effect on the extent of aging delay; this combination included rapamycin and metformin [79, 80]. This notion has also been supported by the following studies in model eukaryotic organisms: 1) pairwise combinations of rapamycin and wortmannin (an inhibitor of phosphoinositide 3-kinase), rapamycin and pyrrolidine dithiocarbamates (PDTC; inhibitors of the NF-κB pathway), and wortmannin and PDTC have been shown to exhibit synergistic effects on the extent of *Drosophila melanogaster* lifespan extension [81]; 2) a pairwise combination of rapamycin and an inhibitor of the stress-activated c-Jun N-terminal kinase have been demonstrated to act in synergy to prolong longevity of the coastal marine and salt-lake rotifer *Brachionus manjavacas* [82]; 3) some double and triple combinations of synthetic drugs and natural chemicals that target the IGF-1, transforming growth factor β and sterol regulatory element-binding protein signaling pathways of longevity regulation have been show to extend the lifespan of the nematode *Caenorhabditis elegans* in a synergistic manner [83]; and 4) rapamycin and myriocin (an inhibitor of sphingolipid synthesis) act in synergy to extend chronological lifespan in the budding yeast *Saccharomyces cerevisiae* and in the fission yeast *Schizosaccharomyces pombe* [84, 85].

Our recent study has revealed six plant extracts (PEs) that significantly extend the chronological lifespan (CLS) of the yeast *S. cerevisiae* under non-CR conditions [86]. We called them PE4 (an extract from the root and rhizome of *Cimicifuga racemosa*), PE5 (an extract from the root of *Valeriana officinalis L.*), PE6 (an extract from the whole plant of *Passiflora incarnate L.*), PE8 (an extract from the leaf of *Ginkgo biloba*), PE12 (an extract from the seed of *Apium graveolens L.*) and PE21 (an extract from the bark of *Salix alba*) [86]. These six longevity-extending PEs are geroprotectors that postpone the onset and decelerate the progression of yeast chronological aging; each of them promotes a hormetic stress response and exhibits a different effect on a distinct set of longevity-defining cellular processes [86]. We have also demonstrated that PE4, PE5, PE6, PE8, PE12 and PE21 delay yeast chronological aging via an evolutionarily conserved network of signaling pathways and protein kinases (Supplementary Figure 1) [87]. Most of the six PEs slows down aging by modulating different nodes, edges and modules of this intricate network of longevity regulation (Supplementary Figure 1) [87]; some of these elements of the network are also under control of such naturally-occurring aging-delaying chemical compounds as spermidine and resveratrol (Supplementary Figure 1) [64, 67, 88–94]. We noticed that, if a strain carrying an aging-delaying single-gene mutation affecting a certain node, edge or module of the network is exposed to some of the six PEs, the mutation and the PE enhance aging-delaying efficiencies of each other so that their combination has a synergistic effect on the extent of aging delay [87]. Based on these observations, we hypothesized that a pairwise combination of two aging-delaying PEs or a combination of one of these PEs and spermidine or resveratrol may exhibit a synergistic effect on the extent of aging delay only if each of the two components of this combination targets a different node, edge or module of the network. This study is a proof-of-concept investigation aimed at testing our hypothesis by a systematic assessment of longevity-extending efficiencies of all possible pairwise combinations of PE4, PE5, PE6, PE8, PE12 and PE21 or of one of these PEs and spermidine or resveratrol in chronologically aging yeast. In support of our hypothesis, we show that only pairwise combinations of naturally-occurring chemical compounds that delay yeast chronological aging through different nodes, edges and modules of the longevity regulation network display synergistic effects on the extent of aging delay. Because investigations in *S. cerevisiae* have previously provided evidence that the major aspects and basic mechanisms of aging and aging-associated pathology are evolutionarily conserved [64, 67, 88, 91, 94–102], this study advances our knowledge of how multicomponent combinations of natural chemical compounds can be used for therapeutic multiplexing of aging delay and longevity extension.

## RESULTS

### Our hypothesis on possible synergistic longevity-extending effects of certain pairwise combinations of the six aging-delaying PEs and/or spermidine and resveratrol

A signaling network that controls the rate of yeast chronological aging is schematically depicted in Supplementary Figure 1. This network integrates the following signaling pathways and protein kinases: 1) the pro-aging TORC1 (target of rapamycin complex 1) pathway; 2) the pro-aging PKA (protein kinase A) pathway; 3) the pro-aging PKH1/2 (Pkb-activating kinase homolog) pathway; 4) the anti-aging SNF1 (sucrose non-fermenting) pathway; 5) the anti-aging ATG (autophagy) pathway; 6) the pro-aging protein kinase Sch9, which is activated by the TORC1 and PKH1/2 pathways; and 7) the anti-aging protein kinase Rim15, which is suppressed by the TORC1, PKA and PKH1/2 pathways (Supplementary Figure 1) [67, 87, 96, 97, 103–107]. The network modulates such longevity-defining cellular processes as gluconeogenesis, glyoxylate cycle, glycogen synthesis and degradation, amino acids synthesis, fatty acids synthesis, mitochondrial respiration, protein synthesis in the cytosol and mitochondria, maintenance of nuclear and mitochondrial genomes, peroxisome biogenesis, autophagy, and stress responses (Supplementary Figure 1) [67, 96, 97, 105–117]. PE4, PE5, PE6, PE8, PE12 and PE21 delay yeast chronological aging because they elicit the following effects on different nodes, edges and modules of the network: 1) PE4 lessens the inhibitory action of the pro-aging TORC1 pathway on the anti-aging SNF1 pathway; 2) PE5 suppresses two different branches of the pro-aging PKA pathway; 3) PE6 regulates cellular processes that are not integrated into the network; 4) PE8 inhibits the suppressive effect of PKA on SNF1; 5) PE12 stimulates the anti-aging protein kinase Rim15; and 6) PE21 downregulates a form of the pro-aging protein kinase Sch9 that is stimulated by the pro-aging PKH1/2 pathway (Supplementary Figure 1) [87].

It has previously demonstrated that spermidine, a polyamine of plant origin, delays the chronological mode of aging in yeast and other organisms by activating the anti-aging ATG1 (autophagy) pathway (Supplementary Figure 1) [88, 89, 92, 97, 100]. Moreover, although resveratrol has been shown to extend mammalian healthspan by suppressing cAMP-dependent phosphodiesterases to elicit AMPK activation [118] and by stimulating the tyrosyl transfer-RNA synthetase to promote poly(ADP-ribose) polymerase 1 auto-poly-ADP-ribosylation [119], the molecular targets of this plant phenolic compound in chronologically aging yeast remain unknown (Supplementary Figure 1).

As we have already mentioned, the following two observations provide the basis for our hypothesis: 1) most of the six aging-delaying PEs, as well as spermidine and resveratrol, modulate different nodes, edges and modules of the signaling network that controls the rate of yeast chronological aging (Supplementary Figure 1) [87]; the only possible exception is the demonstrated abilities of PE4 and PE8 to weaken the restraining action of two different network’s edges (i.e. the pro-aging TORC1 pathway and PKA pathway, respectively) on the same node (i.e. the anti-aging SNF1 pathway) of the network (Supplementary Figure 1) [87]; and 2) certain combinations of one of the six PEs and aging-delaying single-gene mutations that affect these nodes, edges and modules display synergistic effects on the extent of yeast chronological aging delay [87]. We therefore put forward the hypothesis that most of 27 possible pairwise combinations of two aging-delaying PEs or of one of these PEs and spermidine or resveratrol (Supplementary Table 1) may slow down yeast chronological aging in a synergistic manner. We also hypothesized that a combination of PE4 and PE8 may not display a synergistic effect on the extent of yeast chronological aging delay. To test these hypotheses, we assessed longevity-extending efficiencies of all possible pairwise combinations of PE4, PE5, PE6, PE8, PE12 and PE21 or of one of these PEs and spermidine or resveratrol in chronologically aging yeast.

### An effect-based model that we used to assess if a pairwise combination of aging-delaying chemical compounds has a synergistic effect on the extent of aging delay

Several effect-based models have been developed to assess if a pairwise combination of chemical compounds exhibits a synergistic effect on the monitored process, i.e. if the positive effect of this combination on the process exceeds the positive effects of individual compounds comprising the combination [23, 28, 29, 50, 56, 120–123]. In this study, we have used the highest single agent (HSA) model for evaluating if two PEs or a PE and spermidine or resveratrol extend yeast longevity synergistically if used in a pairwise combination; this model has been recently used to demonstrate that certain drug combinations have synergistic effects on aging delay and healthspan extension in the nematode *C. elegans* [83]. According to the HSA model, two chemical compounds are considered to act in synergy if the effect of their combination exceeds the effect of a component of this combination that exhibits the highest effect if it is used alone [50, 56, 120, 123]. Using the HSA model, we have calculated the Combination Index (CI) value (which is considered as the standard measure of combination effect [50, 56, 120, 123]) as follows: CI = CLS_X_/CLS_X+Y_ (if chemical compound X is the HSA) or CI = CLS_Y_/CLS_X+Y_ (if chemical compound Y is the HSA) for both the mean and maximum CLS of yeast exposed to compound X alone, to compound Y alone or to a mixture of compounds X and Y. We have calculated the significance of a synergistic effect (i.e. CI < 1) as the *p* value of the two-tailed *t* test for comparing the effect of a combination of chemical compounds (i.e. CLS_X+Y_) to that of the HSA (i.e. CLS_X_ orCLS_Y_ for the mean and maximum CLS).

### Mixtures of PE4 and PE5, PE4 and PE6, PE4 and PE12, and PE4 and PE21 have synergistic effects on the extent of aging delay

PE4, PE5, PE6, PE12 or PE21 have been shown to modulate different nodes, edges and modules of the signaling network that controls the rate of yeast chronological aging [87]. Specifically, these PEs delay aging as follows: 1) PE4 weakens the restraining action of the pro-aging TORC1 pathway on the anti-aging SNF1 pathway; 2) PE5 mitigates two different branches of the pro-aging PKA pathway; 3) PE6 modulates a presently unknown pro-aging or anti-aging node that may be integrated into this network; 4) PE12 stimulates the anti-aging protein kinase Rim15; and 5) PE21 inhibits a form of the pro-aging protein kinase Sch9 that is activated by the pro-aging PKH1/2 pathway (Supplementary Figure 1) [87]. We therefore hypothesized that mixtures of PE4 with PE5, PE6, PE12 or PE21 may exhibit synergistic effects on the extent of yeast chronological aging delay. To test this hypothesis, we cultured wild-type (WT) cells in the synthetic minimal medium initially containing 2% glucose, either without a PE (i.e. cells were subjected to ethanol-mock treatment) or with the following additions: 1) PE4, PE5, PE6, PE12 or PE21 alone (each being used at the final concentration of 0.1%, 0.5% or 1.0%, see below); or 2) a mixture of 0.1%, 0.3%, 0.5% or 1.0% PE4 with PE5, PE6, PE12 or PE21 (each being used at the final concentration of 0.1%, 0.3%, 0.5% or 1.0%).

**Figure 1:**
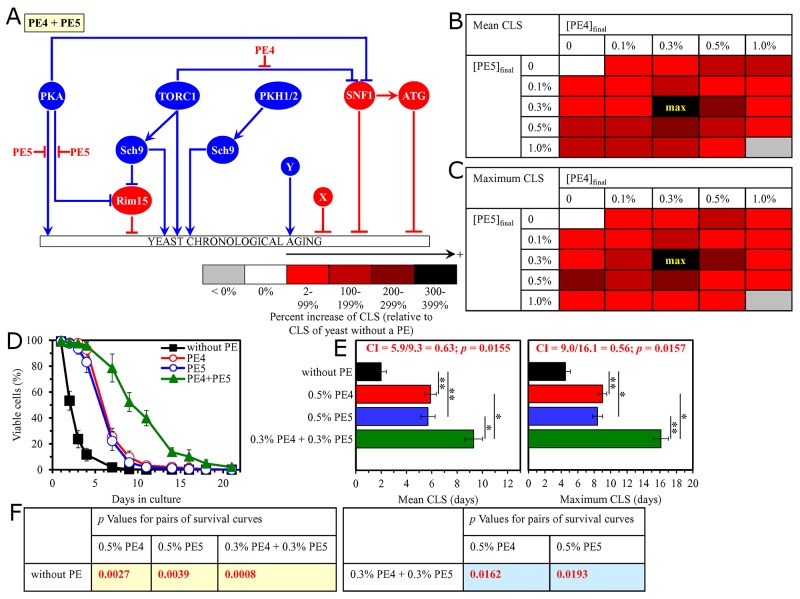
The longevity-extending efficiency of a mixture of 0.3% PE4 and 0.3% PE5 statistically significantly exceeds those of PE4 and PE5, each being used at the optimal concentration of 0.5% Thus, PE4 and PE5 enhance the longevity-extending efficiency of each other. Hence, according to the highest single agent (HSA) model, PE4 and PE5 act in synergy to extend longevity of chronologically aging yeast. **(A)** PE4 and PE5 are known to inhibit different pro-aging nodes of the signaling network that controls the rate of yeast chronological aging. PE4 weakens the restraining action of the pro-aging TORC1 pathway on the anti-aging SNF1 pathway, whereas PE5 mitigates two different branches of the pro-aging PKA pathway. **(B, C)** Wild-type (WT) cells were grown in the synthetic minimal YNB medium initially containing 2% glucose, with PE4 and/or PE5 (at the final concentration of 0.1%, 0.3%, 0.5% or 1.0%) or without a PE. Effects of different concentrations of PE4 and PE5 (added alone or in pairwise combinations) on the mean (B) or maximum (C) chronological lifespan (CLS) of WT cells are shown. The table cell at the intersection of the column for 0.3% PE4 and the row for 0.3% PE5 is marked ″max″ because the mixture of 0.3% PE4 and 0.3% PE5 exhibits the highest extending effect on the mean and maximum lifespans of chronologically aging WT cells. **(D, E)** WT cells were cultured in the synthetic minimal YNB medium initially containing 2% glucose and one of the following supplements: 0.5% PE4, 0.5% PE5, or a mixture of 0.3% PE4 and 0.3% PE5. In the cultures supplemented with PE4 and/or PE5, ethanol was used as a vehicle at the final concentration of 2.5%. In the same experiment, WT cells were also subjected to ethanol-mock treatment by being cultured in the synthetic minimal YNB medium initially containing 2% glucose and 2.5% ethanol. Survival curves (D) and the mean and maximum lifespans (E) of chronologically aging WT cells cultured without a PE (cells were subjected to ethanol-mock treatment), with 0.5% PE4, with 0.5% PE5, or with the mixture of 0.3% PE4 and 0.3% PE5 are shown. Data in D and E are presented as means ± SEM (n = 3; ^*^p < 0.05; ^**^p < 0.01). The Combination Index (CI) values in E were calculated as follows: CI = CLS_PE4_/CLS_PE4+PE5_ for both the mean and maximum CLS; the significance of a synergistic effect (i.e. CI < 1) is provided as the *p* value of the two-tailed *t* test for comparing the effect of a PE combination (i.e. CLS_PE4+PE5_) to that of the HSA (i.e. CLS_PE4_ for both the mean and maximum CLS). Data for mock-treated WT cells are replicated in graphs D and E of Figures 2–14 and Supplementary Figures 2-14. Data for WT cells cultured with 0.5% PE4 are replicated in graphs D and E of Figures 2–4, Figure 11, Supplementary Figure 2 and Supplementary Figure 9. Data for WT cells cultured with 0.5% PE5 are replicated in graphs D and E of Figure 5, Figure 6, Figure 9, Supplementary Figure 3 and Supplementary Figure 4. **(F)** *p* Values for different pairs of survival curves of WT cells cultured in the presence of 0.5% PE4, 0.5% PE5, a mixture of 0.3% PE4 and 0.3% PE5, or in the absence of a PE (cells were subjected to ethanol-mock treatment) are shown. Survival curves shown in (D) were compared. Two survival curves were considered statistically different if the *p* value was less than 0.05. The *p* values for comparing pairs of survival curves using the logrank test were calculated as described in Materials and Methods. The *p* values displayed on a yellow color background indicate that 0.5% PE4, 0.5% PE5, and the mixture of 0.3% PE4 and 0.3% PE5 significantly extend the CLS of WT cells. The *p* values displayed on a blue color background indicate that the CLS-extending efficiency of the mixture of 0.3% PE4 and 0.3% PE5 significantly exceeds that of 0.5% PE4 or 0.5% PE5. Abbreviations: ATG, autophagy; PKA, protein kinase A; PKH1/2, Pkb-activating kinase homologs 1 and 2; Rim15, an anti-aging protein kinase; Sch9, a pro-aging protein kinase; SNF1, sucrose non-fermenting protein 1; TORC1, target of rapamycin complex 1; X, a presently unknown anti-aging node of this signaling network; Y, a presently unknown pro-aging node of this signaling network.

**Figure 3:**
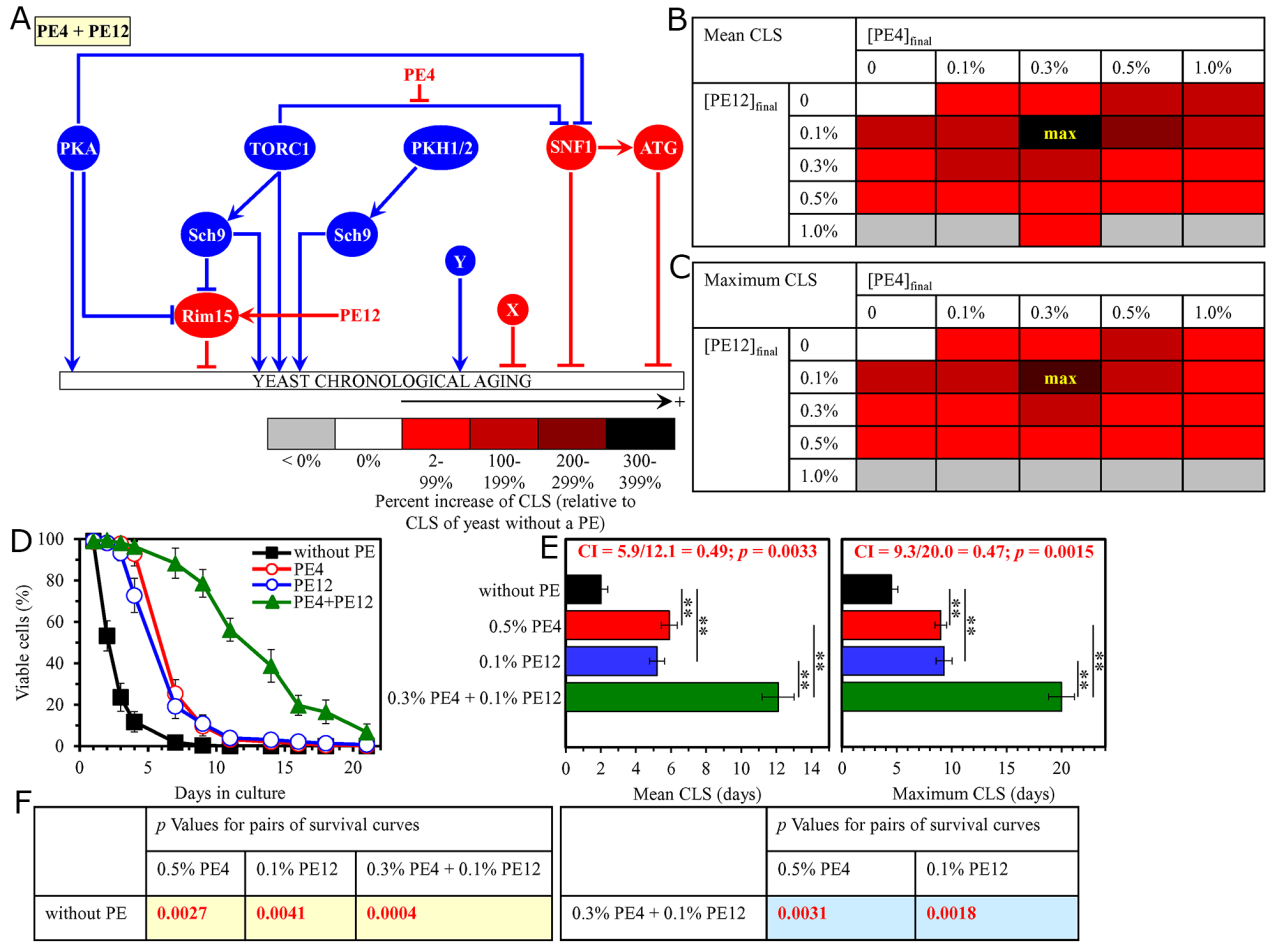
The longevity-extending efficiency of a mixture of 0.3% PE4 and 0.1% PE12 statistically significantly exceeds those of PE4 and PE12, which were used at the optimal concentration of 0.5% or 0.1% (respectively) Thus, PE4 and PE12 enhance the longevity-extending efficiency of each other. Hence, according to the HSA model, PE4 and PE12 act in synergy to extend longevity of chronologically aging yeast. **(A)** PE4 and PE12 are known to inhibit different pro-aging nodes of the signaling network that controls the rate of yeast chronological aging. PE4 weakens the restraining action of the pro-aging TORC1 pathway on the anti-aging SNF1 pathway, whereas PE12 stimulates the anti-aging protein kinase Rim15. **(B, C)** WT cells were grown as described in the legend to Figure 1, with PE4 and/or PE12 (at the final concentration of 0.1%, 0.3%, 0.5% or 1.0%) or without a PE. Effects of different concentrations of PE4 and PE12 (added alone or in pairwise combinations) on the mean (B) or maximum (C) CLS of WT cells are shown. The table cell at the intersection of the column for 0.3% PE4 and the row for 0.1% PE12 is marked ″max″ for the reason described in the legend to Figure 1. **(D, E)** WT cells were cultured as described in the legend to Figure 1, with one of the following supplements: 0.5% PE4, 0.1% PE12, or a mixture of 0.3% PE4 and 0.1% PE12. Ethanol was used as a vehicle or for mock treatment as described in the legend to Figure 1. Survival curves (D) and the mean and maximum lifespans (E) of chronologically aging WT cells cultured without a PE (cells were subjected to ethanol-mock treatment), with 0.5% PE4, with 0.1% PE12, or with the mixture of 0.3% PE4 and 0.1% PE12 are shown. Data in D and E are presented as means ± SEM (n = 3; ^*^p < 0.05; ^**^p < 0.01). The CI and *p* values in E were calculated as described in the legend to Figure 1. Data for mock-treated WT cells are replicated in graphs D and E of Figure 1, Figure 2, Figures 4–14 and Supplementary Figures 2-14. Data for WT cells cultured with 0.5% PE4 are replicated in graphs D and E of Figure 1, Figure 2, Figure 4, Figure 11, Supplementary Figure 2 and Supplementary Figure 9. Data for WT cells cultured with 0.1% PE12 are replicated in graphs D and E of Figure 7, Figure 8, Figure 13, Supplementary Figure 4, Supplementary Figure 8 and Supplementary Figure 11. **(F)** *p* Values for different pairs of survival curves of WT cells cultured in the presence of 0.5% PE4, 0.1% PE12, a mixture of 0.3% PE4 and 0.1% PE12, or in the absence of a PE (cells were subjected to ethanol-mock treatment) are shown. Survival curves shown in (D) were compared. The *p* values are displayed on a yellow or blue color background for the reasons described in the legend to Figure 1. Abbreviations: as in the legend to Figure 1.

**Figure 4:**
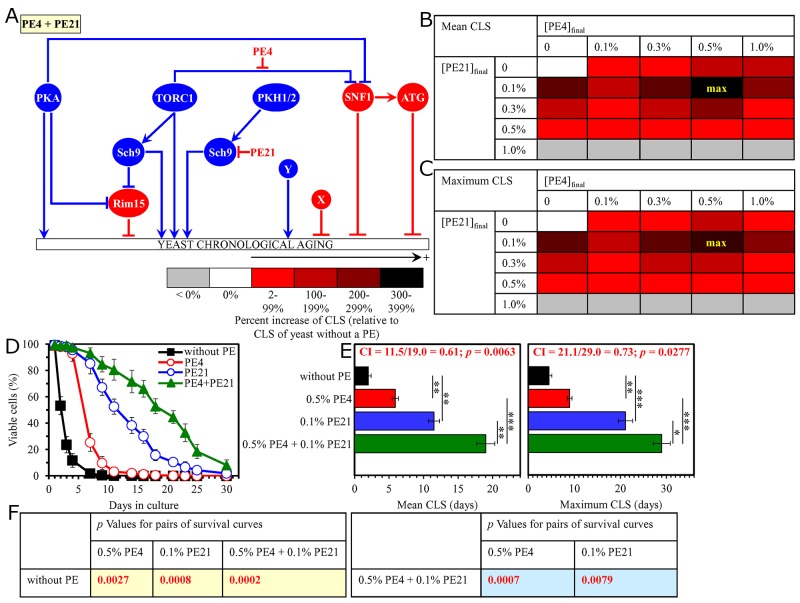
The longevity-extending efficiency of a mixture of 0.5% PE4 and 0.1% PE21 statistically significantly exceeds those of PE4 and PE21, which were used at the optimal concentration of 0.5% or 0.1% (respectively) Thus, PE4 and PE21 enhance the longevity-extending efficiency of each other. Hence, according to the HSA model, PE4 and PE21 act in synergy to extend longevity of chronologically aging yeast. **(A)** PE4 and PE21 are known to inhibit different pro-aging nodes of the signaling network that controls the rate of yeast chronological aging. PE4 weakens the restraining action of the pro-aging TORC1 pathway on the anti-aging SNF1 pathway, whereas PE21 mitigates a form of the pro-aging protein kinase Sch9 that is activated by the pro-aging PKH1/2 pathway. **(B, C)** WT cells were grown as described in the legend to Figure 1, with PE4 and/or PE21 (at the final concentration of 0.1%, 0.3%, 0.5% or 1.0%) or without a PE. Effects of different concentrations of PE4 and PE21 (added alone or in pairwise combinations) on the mean (B) or maximum (C) CLS of WT cells are shown. The table cell at the intersection of the column for 0.5% PE4 and the row for 0.1% PE21 is marked ″max″ for the reason described in the legend to Figure 1. **(D, E)** WT cells were cultured as described in the legend to Figure 1, with one of the following supplements: 0.5% PE4, 0.1% PE21, or a mixture of 0.5% PE4 and 0.1% PE21. Ethanol was used as a vehicle or for mock treatment as described in the legend to Figure 1. Survival curves (D) and the mean and maximum lifespans (E) of chronologically aging WT cells cultured without a PE (cells were subjected to ethanol-mock treatment), with 0.5% PE4, with 0.1% PE21, or with the mixture of 0.5% PE4 and 0.1% PE21 are shown. Data in D and E are presented as means ± SEM (n = 3; ^*^p < 0.05; ^**^p < 0.01; ^***^ < 0.001). The CI and *p* values in E were calculated as described in the legend to Figure 1. Data for mock-treated WT cells are replicated in graphs D and E of Figures 1–3, Figure 5, Figures 6–14 and Supplementary Figures 2-14. Data for WT cells cultured with 0.5% PE4 are replicated in graphs D and E of Figures 1–3, Figure 11, Supplementary Figure 2 and Supplementary Figure 9. Data for WT cells cultured with 0.1% PE21 are replicated in graphs D and E of Figure 6, Figure 14, Supplementary Figures 6-8 and Supplementary Figure 12. **(F)** *p* Values for different pairs of survival curves of WT cells cultured in the presence of 0.5% PE4, 0.1% PE21, a mixture of 0.5% PE4 and 0.1% PE21, or in the absence of a PE (cells were subjected to ethanol-mock treatment) are shown. Survival curves shown in (D) were compared. The *p* values are displayed on a yellow or blue color background for the reasons described in the legend to Figure 1. Abbreviations: as in the legend to Figure 1.

**Figure 2:**
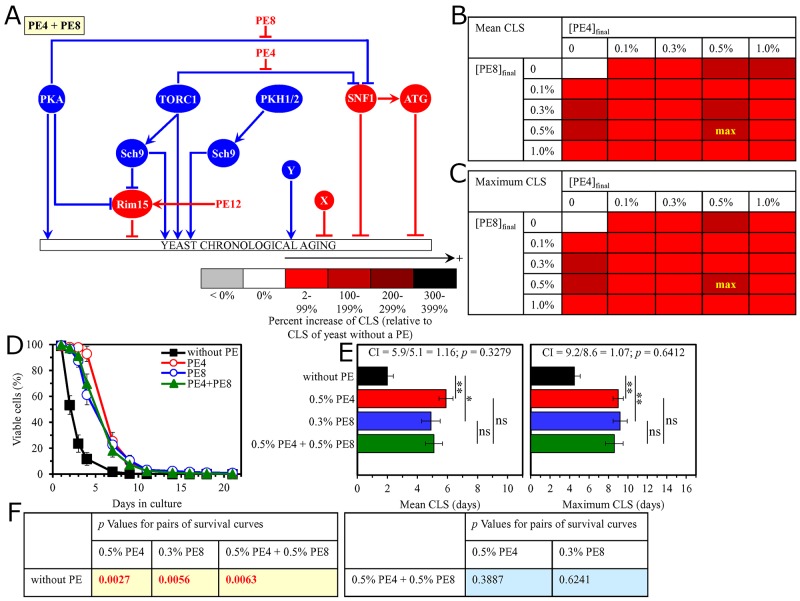
The longevity-extending efficiency of a mixture of 0.5% PE4 and 0.5% PE8 is not statistically different from those of PE4 and PE8, which were used at the optimal concentration of 0.5% or 0.3% (respectively) Thus, PE4 and PE8 do not enhance the longevity-extending efficiency of each other. Hence, according to the HSA model, PE4 and PE8 do not act in synergy to extend longevity of chronologically aging yeast. **(A)** PE4 and PE8 are known to weaken the restraining action of the pro-aging TORC1 or PKA pathway on the same node (i.e. the anti-aging SNF1 pathway) of the signaling network that controls the rate of yeast chronological aging. **(B, C)** WT cells were grown as described in the legend to Figure 1, with PE4 and/or PE8 (at the final concentration of 0.1%, 0.3%, 0.5% or 1.0%) or without a PE. Effects of different concentrations of PE4 and PE8 (added alone or in pairwise combinations) on the mean (B) or maximum (C) CLS of WT cells are shown. The table cell at the intersection of the column for 0.5% PE4 and the row for 0.5% PE8 is marked ″max″ for the reason described in the legend to Figure 1. **(D, E)** WT cells were cultured as described in the legend to Figure 1, with one of the following supplements: 0.5% PE4, 0.3% PE8, or a mixture of 0.5% PE4 and 0.5% PE8. Ethanol was used as a vehicle or for mock treatment as described in the legend to Figure 1. Survival curves (D) and the mean and maximum lifespans (E) of chronologically aging WT cells cultured without a PE (cells were subjected to ethanol-mock treatment), with 0.5% PE4, with 0.3% PE8, or with the mixture of 0.5% PE4 and 0.5% PE8 are shown. Data in D and E are presented as means ± SEM (n = 3; ^*^p < 0.05; ^**^p < 0.01). The CI and *p* values in E were calculated as described in the legend to Figure 1. Data for mock-treated WT cells are replicated in graphs D and E of Figure 1, Figures 3–14 and Supplementary Figures 2-14. Data for WT cells cultured with 0.5% PE4 are replicated in graphs D and E of Figure 1, Figure 3, Figure 4, Figure 11, Supplementary Figure 2 and Supplementary Figure 9. Data for WT cells cultured with 0.3% PE8 are replicated in graphs D and E of Figure 5, Figure 8, Figure 10, Supplementary Figure 5, Supplementary Figure 7 and Supplementary Figure 14. **(F)** *p* Values for different pairs of survival curves of WT cells cultured in the presence of 0.5% PE4, 0.3% PE8, a mixture of 0.5% PE4 and 0.5% PE8, or in the absence of a PE (cells were subjected to ethanol-mock treatment) are shown. Survival curves shown in (D) were compared. Abbreviations: as in the legend to Figure 1.

We found that the longevity-extending efficiencies of the following pairwise combinations of aging-delaying PEs statistically significantly exceed that of a PE within the pair which was considered as the HSA if this PE was used alone at the optimal aging-delaying concentration: 1) a mixture of 0.3% PE4 and 0.3% PE5 (if PE4 and PE5 were used at these final concentrations, their mixture exhibited the highest longevity-extending effect) as compared to 0.5% PE4 (which was used as the HSA for both the mean and maximum CLS), with CI = 0.63 for the mean CLS and CI = 0.56 for the maximum CLS (Figure 1); 2) a mixture of 0.5% PE4 and 1.0% PE6 as compared to 0.5% PE4 (which was used as the HSA for both the mean and maximum CLS) (Supplementary Figure 2); 3) a mixture of 0.5% PE4 and 0.1% PE12 as compared to 0.5% PE4 (which was used as the HSA for the mean CLS) or 0.1% PE12 (which was used as the HSA for the maximum CLS) (Figure 3); and 4) a mixture of 0.5% PE4 and 0.1% PE21 as compared to 0.1% PE21 (which was used as the HSA for both the mean and maximum CLS), with CI = 0.61 for the mean CLS and CI = 0.73 for the maximum CLS (Figure 4).

In sum, these findings confirm our hypothesis that mixtures of PE4 with PE5, PE6, PE12 or PE21 slow down yeast chronological aging in a synergistic manner.

### A mixture of PE4 and PE8 does not slow down yeast chronological aging in a synergistic manner

PE4 and PE8 have been shown to weaken the restraining action of the pro-aging TORC1 or PKA pathway on the same node (i.e. the anti-aging SNF1 pathway) of the signaling network that controls the rate of yeast chronological aging (Supplementary Figure 1) [87]. We therefore hypothesized that PE4 and PE8 may not act in synergy to delay yeast chronological aging. To test this hypothesis, we cultured WT cells in the synthetic minimal medium initially containing 2% glucose, either without a PE (i.e. cells were subjected to ethanol-mock treatment) or with the following additions: 1) 0.5% PE4 or 0.3% PE8 alone (if PE4 or PE8 was used at this final concentration, it exhibited the highest longevity-extending effect); or 2) a mixture of 0.1%, 0.3%, 0.5% or 1.0% PE4 with 0.1%, 0.3%, 0.5% or 1.0% PE8).

In support of our hypothesis, we found that the longevity-extending efficiency of a mixture of 0.5% PE4 and 0.5% PE8 is not statistically different from those of PE4 and PE8, which were used at the optimal concentration of 0.5% or 0.3% (respectively) (Figure 2). The CI values were 1.16 and 1.07 for the mean CLS and the maximum CLS (respectively) when 0.5% PE4 was used as the HSA for the mean CLS and 0.3% PE8 was used as the HSA for the maximum CLS (Figure 2).

### Pairwise combinations of PE5 and PE6, PE5 and PE8, PE5 and PE12, and PE5 and PE21 delay yeast chronological aging in a synergistic fashion

As we have already mentioned, PE5, PE6, PE8, PE12 and PE21 are known to affect different nodes, edges and modules of the signaling network that controls the rate of yeast chronological aging (Supplementary Figure 1) [87]. We therefore put forward the hypothesis that pairwise combinations of PE5 and PE6, PE5 and PE8, PE5 and PE12, and PE5 and PE21 may synergistically extend longevity of chronologically aging yeast. To test this hypothesis, WT cells were cultured in the synthetic minimal medium initially containing 2% glucose, either without a PE (i.e. cells were subjected to ethanol-mock treatment) or with the following additions: 1) PE5, PE6, PE8, PE12 or PE21 alone (each being used at the final concentration of 0.1%, 0.3%, 0.5% or 1.0%, see below); or 2) a pairwise combination of 0.1%, 0.3%, 0.5% or 1.0% PE5 and PE6, PE8, PE12 or PE21 (each being used at the final concentration of 0.1%, 0.3%, 0.5% or 1.0%).

**Figure 5:**
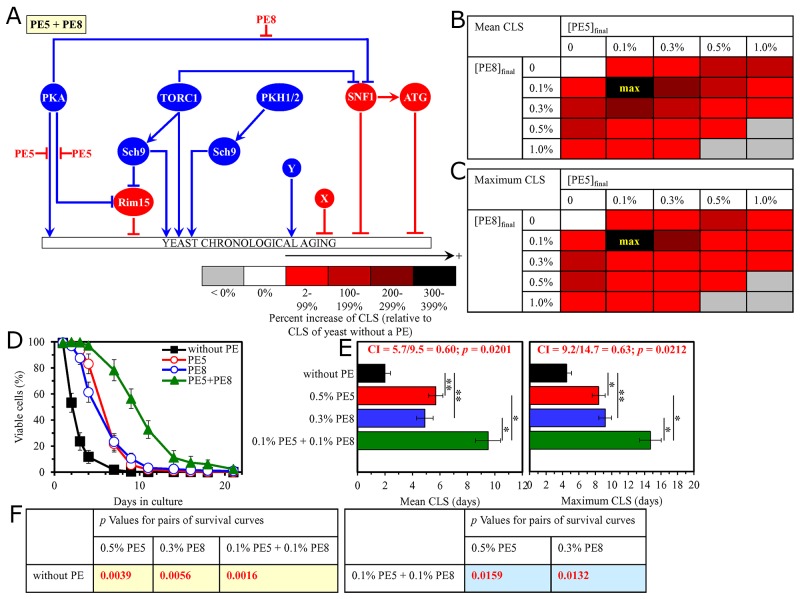
The longevity-extending efficiency of a mixture of 0.1% PE5 and 0.1% PE8 statistically significantly exceeds those of PE5 and PE8, which were used at the optimal concentration of 0.5% or 0.3% (respectively) Thus, PE5 and PE8 enhance the longevity-extending efficiency of each other. Hence, according to the HSA model, PE5 and PE8 act in synergy to extend longevity of chronologically aging yeast. **(A)** PE5 and PE8 are known to inhibit different pro-aging nodes of the signaling network that controls the rate of yeast chronological aging. PE5 mitigates two different branches of the pro-aging PKA pathway, whereas PE8 weakens the restraining action of this pathway on the anti-aging SNF1 pathway. **(B, C)** WT cells were grown as described in the legend to Figure 1, with PE5 and/or PE8 (at the final concentration of 0.1%, 0.3%, 0.5% or 1.0%) or without a PE. Effects of different concentrations of PE5 and PE8 (added alone or in pairwise combinations) on the mean (B) or maximum (C) CLS of WT cells are shown. The table cell at the intersection of the column for 0.1% PE5 and the row for 0.1% PE8 is marked ″max″ for the reason described in the legend to Figure 1. **(D, E)** WT cells were cultured as described in the legend to Figure 1, with one of the following supplements: 0.5% PE5, 0.3% PE8, or a mixture of 0.1% PE5 and 0.1% PE8. Ethanol was used as a vehicle or for mock treatment as described in the legend to Figure 1. Survival curves (D) and the mean and maximum lifespans (E) of chronologically aging WT cells cultured without a PE (cells were subjected to ethanol-mock treatment), with 0.5% PE5, with 0.3% PE8, or with the mixture of 0.1% PE5 and 0.1% PE8 are shown. Data in D and E are presented as means ± SEM (n = 3; ^*^p < 0.05; ^**^p < 0.01). The CI and *p* values in E were calculated as described in the legend to Figure 1. Data for mock-treated WT cells are replicated in graphs D and E of Figures 1–4, Figures 6–14 and Supplementary Figures 2-14. Data for WT cells cultured with 0.5% PE5 are replicated in graphs D and E of Figure 1, Figure 6, Figure 9, Supplementary Figure 3 and Supplementary Figure 4. Data for WT cells cultured with 0.3% PE8 are replicated in graphs D and E of Figure 2, Figure 8, Figure 10, Supplementary Figure 5, Supplementary Figure 7 and Supplementary Figure 14. **(F)** *p* Values for different pairs of survival curves of WT cells cultured in the presence of 0.5% PE5, 0.3% PE8, a mixture of 0.1% PE5 and 0.1% PE8, or in the absence of a PE (cells were subjected to ethanol-mock treatment) are shown. Survival curves shown in (D) were compared. The *p* values are displayed on a yellow or blue color background for the reasons described in the legend to Figure 1. Abbreviations: as in the legend to Figure 1.

**Figure 6:**
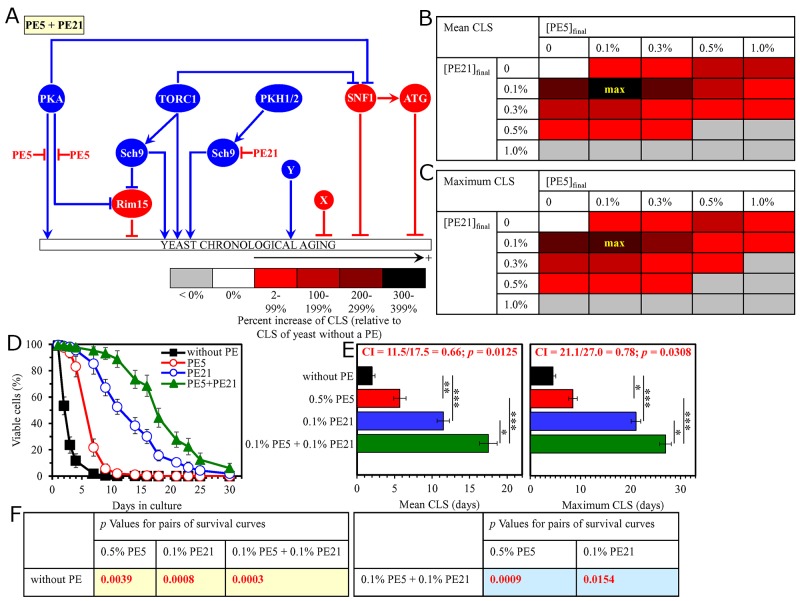
The longevity-extending efficiency of a mixture of 0.1% PE5 and 0.1% PE21 statistically significantly exceeds those of PE5 and PE21, which were used at the optimal concentration of 0.5% or 0.1% (respectively) Thus, PE5 and PE21 enhance the longevity-extending efficiency of each other. Hence, according to the HSA model, PE5 and PE21 act in synergy to extend longevity of chronologically aging yeast. **(A)** PE5 and PE21 are known to inhibit different pro-aging nodes of the signaling network that controls the rate of yeast chronological aging. PE5 mitigates two different branches of the pro-aging PKA pathway, whereas PE21 mitigates a form of the pro-aging protein kinase Sch9 that is activated by the pro-aging PKH1/2 pathway. **(B, C)** WT cells were grown as described in the legend to Figure 1, with PE5 and/or PE21 (at the final concentration of 0.1%, 0.3%, 0.5% or 1.0%) or without a PE. Effects of different concentrations of PE5 and PE21 (added alone or in pairwise combinations) on the mean (B) or maximum (C) CLS of WT cells are shown. The table cell at the intersection of the column for 0.1% PE5 and the row for 0.1% PE21 is marked ″max″ for the reason described in the legend to Figure 1. **(D, E)** WT cells were cultured as described in the legend to Figure 1, with one of the following supplements: 0.5% PE5, 0.1% PE21, or a mixture of 0.1% PE5 and 0.1% PE21. Ethanol was used as a vehicle or for mock treatment as described in the legend to Figure 1. Survival curves (D) and the mean and maximum lifespans (E) of chronologically aging WT cells cultured without a PE (cells were subjected to ethanol-mock treatment), with 0.5% PE5, with 0.1% PE21, or with the mixture of 0.1% PE5 and 0.1% PE21 are shown. Data in D and E are presented as means ± SEM (n = 3; ^*^p < 0.05; ^**^p < 0.01; ^***^ < 0.001). The CI and *p* values in E were calculated as described in the legend to Figure 1. Data for mock-treated WT cells are replicated in graphs D and E of Figures 1–5, Figures 7–14 and Supplementary Figures 2-14. Data for WT cells cultured with 0.5% PE5 are replicated in graphs D and E of Figure 1, Figure 5, Figure 9, Supplementary Figure 3 and Supplementary Figure 4. Data for WT cells cultured with 0.1% PE21 are replicated in graphs D and E of Figure 4, Figure 14, Supplementary Figures 6-8 and Supplementary Figure 12. **(F)** *p* Values for different pairs of survival curves of WT cells cultured in the presence of 0.5% PE5, 0.1% PE21, a mixture of 0.1% PE5 and 0.1% PE21, or in the absence of a PE (cells were subjected to ethanol-mock treatment) are shown. Survival curves shown in (D) were compared. The *p* values are displayed on a yellow or blue color background for the reasons described in the legend to Figure 1. Abbreviations: as in the legend to Figure 1.

We found that the longevity-extending efficiencies of the following pairwise combinations of aging-delaying PEs are statistically significantly greater than that of a PE component of the pair which was used as the HSA if added alone at the concentration exhibiting the highest aging-delaying effect: 1) a pairwise combination of 0.3% PE5 and 0.3% PE6 as compared to 0.5% PE5 (which was used as the HSA for both the mean and maximum CLS) (Supplementary Figure 3); 2) a pairwise combination of 0.1% PE5 and 0.1% PE8 as compared to 0.5% PE5 (which was considered as the HSA for the mean CLS) or 0.3% PE8 (which was considered as the HSA for the maximum CLS) (Figure 5); 3) a pairwise combination of 0.1% PE5 and 0.1% PE12 as compared to 0.5% PE5 (which was used as the HSA for the mean CLS) or 0.1% PE12 (which was used as the HSA for the maximum CLS) (Supplementary Figure 4); and 4) a pairwise combination of 0.1% PE5 and 0.1% PE21 as compared to 0.1% PE21 (which was used as the HSA for both the mean and maximum CLS) (Figure 6).

These findings confirm our hypothesis that pairwise combinations of PE5 and PE6, PE5 and PE8, PE5 and PE12, and PE5 and PE21 synergistically extend longevity of chronologically aging yeast.

### Mixtures of PE6 and PE8, PE6 and PE12, and PE6 and PE21 synergistically extend longevity of chronologically aging yeast

Because PE6, PE8, PE12 and PE21 are known to affect different nodes, edges and modules of the signaling network that controls the rate of yeast chronological aging (Supplementary Figure 1) [87], we hypothesized that mixtures of PE6 with PE8, PE12 or PE21 may have synergistic effects on the efficiency of aging delay. To test this hypothesis, we cultured WT cells in the synthetic minimal medium initially containing 2% glucose, either without a PE (i.e. cells were subjected to ethanol-mock treatment) or with the following additions: 1) PE6, PE8, PE12 or PE21 alone (each being used at the final concentration of 0.1%, 0.3% or 1.0%, see below); or 2) a mixture of 0.1%, 0.3%, 0.5% or 1.0% PE6 with PE8, PE12 or PE21 (each being used at the final concentration of 0.1%, 0.3%, 0.5% or 1.0%).

**Figure 7:**
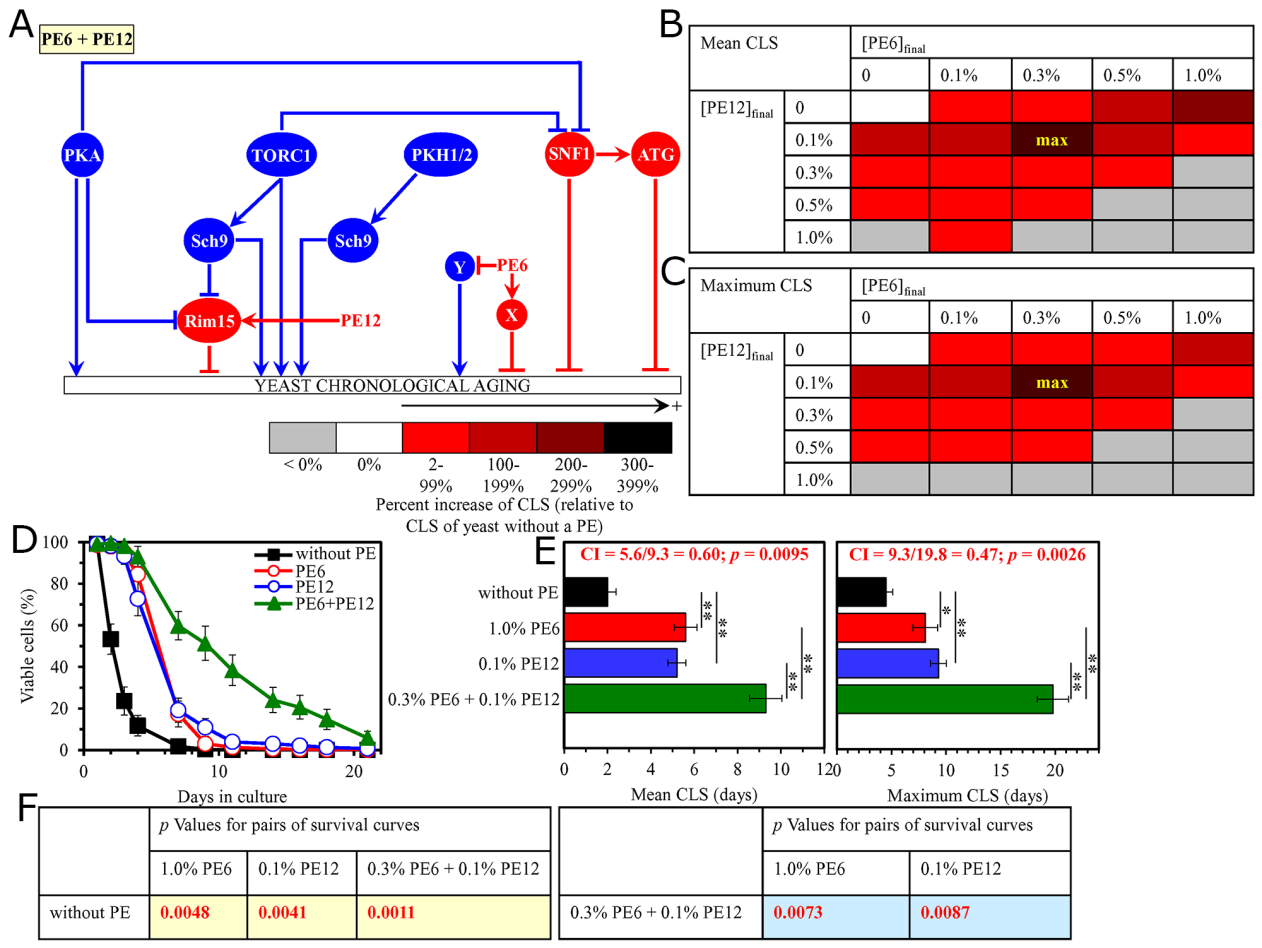
The longevity-extending efficiency of a mixture of 0.3% PE6 and 0.1% PE12 statistically significantly exceeds those of PE6 and PE12, which were used at the optimal concentration of 1.0% or 0.1% (respectively) Thus, PE6 and PE12 enhance the longevity-extending efficiency of each other. Hence, according to the HSA model, PE6 and PE12 act in synergy to extend longevity of chronologically aging yeast. **(A)** PE6 and PE12 are known to regulate different nodes of the signaling network that controls the rate of yeast chronological aging. PE12 stimulates the anti-aging protein kinase Rim15 assimilated into this signaling network, whereas PE6 modulates a presently unknown pro-aging or anti-aging node that may be integrated into this network. **(B, C)** WT cells were grown as described in the legend to Figure 1, with PE6 and/or PE12 (at the final concentration of 0.1%, 0.3%, 0.5% or 1.0%) or without a PE. Effects of different concentrations of PE6 and PE12 (added alone or in pairwise combinations) on the mean (B) or maximum (C) CLS of WT cells are shown. The table cell at the intersection of the column for 0.3% PE6 and the row for 0.1% PE12 is marked ″max″ for the reason described in the legend to Figure 1. **(D, E)** WT cells were cultured as described in the legend to Figure 1, with one of the following supplements: 1.0% PE6, 0.1% PE12, or a mixture of 0.3% PE6 and 0.1% PE12. Ethanol was used as a vehicle or for mock treatment as described in the legend to Figure 1. Survival curves (D) and the mean and maximum lifespans (E) of chronologically aging WT cells cultured without a PE (cells were subjected to ethanol-mock treatment), with 1.0% PE6, with 0.1% PE12, or with the mixture of 0.3% PE6 and 0.1% PE12 are shown. Data in D and E are presented as means ± SEM (n = 3; ^*^p < 0.05; ^**^p < 0.01). The CI and *p* values in E were calculated as described in the legend to Figure 1. Data for mock-treated WT cells are replicated in graphs D and E of Figures 1–6, Figures 8–14 and Supplementary Figures 2-14. Data for WT cells cultured with 1.0% PE6 are replicated in graphs D and E of Figure 12, Supplementary Figure 2, Supplementary Figure 3, Supplementary Figure 5, Supplementary Figure 6 and Supplementary Figure 10. Data for WT cells cultured with 0.1% PE12 are replicated in graphs D and E of Figure 3, Figure 8, Figure 13, Supplementary Figure 4, Supplementary Figure 8 and Supplementary Figure 11. **(F)** *p* Values for different pairs of survival curves of WT cells cultured in the presence of 1.0% PE6, 0.1% PE12, a mixture of 0.3% PE6 and 0.1% PE12, or in the absence of a PE (cells were subjected to ethanol-mock treatment) are shown. Survival curves shown in (D) were compared. The *p* values displayed on a yellow color background indicate that 1.0% PE6, 0.1% PE12, and the mixture of 0.3% PE6 and 0.1% PE12 significantly extend the CLS of WT cells. The *p* values are displayed on a yellow or blue color background for the reasons described in the legend to Figure 1. Abbreviations: as in the legend to Figure 1.

We found that the longevity-extending efficiencies of the following mixtures of aging-delaying PEs are statistically significantly greater than that of a PE component of the mixture which was considered as the HSA if used alone at the concentration having the maximum aging-delaying effect: 1) a mixture of 0.3% PE6 and 0.3% PE8 as compared to 1.0% PE6 (which was considered as the HSA for the mean CLS) or 0.3% PE8 (which was considered as the HSA for the maximum CLS) (Supplementary Figure 5); 2) a mixture of 0.3% PE6 and 0.1% PE12 as compared to 1.0% PE6 (which was used as the HSA for the mean CLS) or 0.1% PE12 (which was used as the HSA for the maximum CLS) (Figure 7); and 3) a mixture of 0.1% PE6 and 0.1% PE21 as compared to 0.1% PE21 (which was considered as the HSA for both the mean and maximum CLS) (Supplementary Figure 6).

Thus, in support of our hypothesis, mixtures of PE6 with PE8, PE12 or PE21 delay yeast chronological aging in a synergistic fashion.

### Pairwise combinations of PE8 with PE12 or PE21 synergistically slow down yeast chronological aging

As has been noted above, PE8, PE12 and PE21 modulate different nodes, edges and modules of the signaling network that defines the rate of yeast chronological aging (Supplementary Figure 1) [87]. We therefore put forward the hypothesis that pairwise combinations of PE8 and PE12, and PE8 and PE21 may synergistically prolong longevity of chronologically aging yeast. To test this hypothesis, WT cells were cultured in the synthetic minimal medium initially containing 2% glucose, either without a PE (i.e. cells were subjected to ethanol-mock treatment) or with the following additions: 1) PE8, PE12 or PE21 alone (each being used at the final concentration of 0.1% or 0.3%, see below); or 2) a pairwise combination of 0.1%, 0.3%, 0.5% or 1.0% PE8 and PE12 or PE21 (each being used at the final concentration of 0.1%, 0.3%, 0.5% or 1.0%).

**Figure 8:**
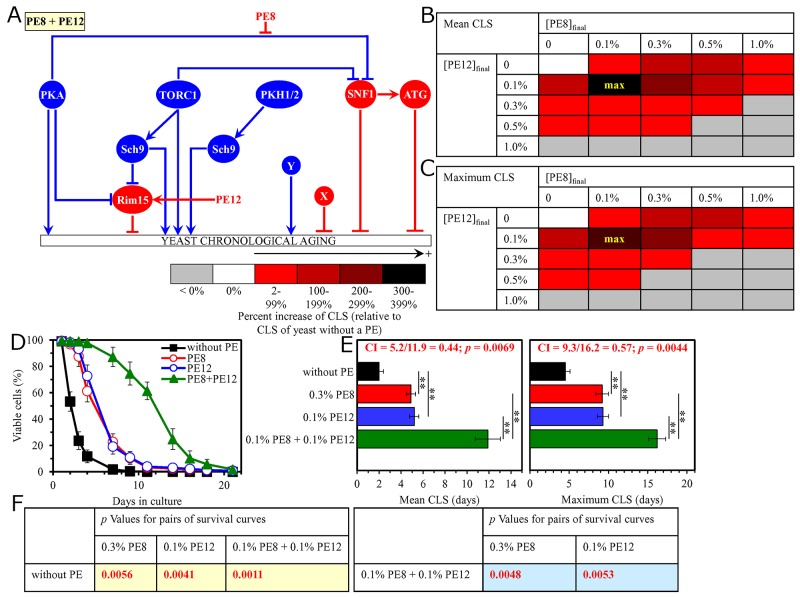
The longevity-extending efficiency of a mixture of 0.1% PE8 and 0.1% PE12 statistically significantly exceeds those of PE8 and PE12, which were used at the optimal concentration of 0.3% or 0.1% (respectively) Thus, PE8 and PE12 enhance the longevity-extending efficiency of each other. Hence, according to the HSA model, PE8 and PE12 act in synergy to extend longevity of chronologically aging yeast. **(A)** PE8 and PE12 are known to regulate different nodes of the signaling network that controls the rate of yeast chronological aging. PE8 weakens the restraining action of the pro-aging PKA pathway on the anti-aging SNF1 pathway, whereas PE12 stimulates the anti-aging protein kinase Rim15 integrated into this signaling network. **(B, C)** WT cells were grown as described in the legend to Figure 1, with PE8 and/or PE12 (at the final concentration of 0.1%, 0.3%, 0.5% or 1.0%) or without a PE. Effects of different concentrations of PE8 and PE12 (added alone or in pairwise combinations) on the mean (B) or maximum (C) CLS of WT cells are shown. The table cell at the intersection of the column for 0.1% PE8 and the row for 0.1% PE12 is marked ″max″ for the reason described in the legend to Figure 1. **(D, E)** WT cells were cultured as described in the legend to Figure 1, with one of the following supplements: 0.3% PE8, 0.1% PE12, or a mixture of 0.1% PE8 and 0.1% PE12. Ethanol was used as a vehicle or for mock treatment as described in the legend to Figure 1. Survival curves (D) and the mean and maximum lifespans (E) of chronologically aging WT cells cultured without a PE (cells were subjected to ethanol-mock treatment), with 0.3% PE8, with 0.1% PE12, or with the mixture of 0.1% PE8 and 0.1% PE12 are shown. Data in D and E are presented as means ± SEM (n = 3; ^**^p < 0.01). The CI and *p* values in E were calculated as described in the legend to Figure 1. Data for mock-treated WT cells are replicated in graphs D and E of Figures 1–7, Figures 9–14 and Supplementary Figures 2-14. Data for WT cells cultured with 0.3% PE8 are replicated in graphs D and E of Figure 2, Figure 5, Figure 10, Supplementary Figure 5, Supplementary Figure 7 and Supplementary Figure 14. Data for WT cells cultured with 0.1% PE12 are replicated in graphs D and E of Figure 3, Figure 7, Figure 13, Supplementary Figure 4, Supplementary Figure 8 and Supplementary Figure 11. **(F)** *p* Values for different pairs of survival curves of WT cells cultured in the presence of 0.3% PE8, 0.1% PE12, a mixture of 0.1% PE8 and 0.1% PE12, or in the absence of a PE (cells were subjected to ethanol-mock treatment) are shown. Survival curves shown in (D) were compared. The *p* values are displayed on a yellow or blue color background for the reasons described in the legend to Figure 1. Abbreviations: as in the legend to Figure 1.

We found that the longevity-extending efficiencies of the following pairwise combinations of aging-delaying PEs statistically significantly exceed that of a PE component of the pair which was considered as the HSA if added alone at the concentration displaying the highest aging-delaying effect: 1) a pairwise combination of 0.1% PE8 and 0.1% PE12 as compared to 0.1% PE12 (which was considered as the HSA for both the mean and maximum CLS) (Figure 8); and 2) a pairwise combination of 0.1% PE8 and 0.1% PE21 as compared to 0.1% PE21 (which was used as the HSA for both the mean and maximum CLS) (Supplementary Figure 7).

Taking together, these findings confirm our hypothesis that pairwise combinations of PE8 with PE12 or PE21 synergistically prolong longevity of chronologically aging yeast.

### A mixture of PE12 and PE21 slows down yeast chronological aging in a synergistic manner

Because PE12 and PE21 are known to affect two different nodes of the signaling network that controls the rate of yeast chronological aging (Supplementary Figure 1) [87], we hypothesized that PE12 and PE21 may not act in synergy to delay yeast chronological aging. To test this hypothesis, we cultured WT cells in the synthetic minimal medium initially containing 2% glucose, either without a PE (i.e. cells were subjected to ethanol-mock treatment) or with the following additions: 1) 0.1% PE12 or 0.1% PE21 alone (if PE12 or PE21 was used at this final concentration, it had the greatest longevity-extending effect); or 2) a mixture of 0.1%, 0.3%, 0.5% or 1.0% PE12 with 0.1%, 0.3%, 0.5% or 1.0% PE21).

In support of our hypothesis, we found that the longevity-extending efficiency of a mixture of 0.1% PE12 and 0.1% PE21 is statistically significantly greater than that of 0.1% PE21 (which was considered as the HSA if used alone at this concentration to attain the maximum aging-delaying effect) (Supplementary Figure 8).

### Pairwise combinations of spermidine with PE4, PE5, PE6, PE8, PE12 or PE21 have synergistic effects on the extent of aging delay

Spermidine has been shown to delay yeast chronological aging by activating the anti-aging ATG1 pathway (Supplementary Figure 1) [88, 89, 92, 97, 100]. Neither PE4, PE5, PE6, PE8, PE12 nor PE21 affects the ATG1 node of the signaling network that defines the rate of yeast chronological aging (Supplementary Figure 1) [87]. We therefore hypothesized that pairwise combinations of spermidine with PE4, PE5, PE6, PE8, PE12 or PE21 may exhibit synergistic effects on the extent of yeast chronological aging delay. To test this hypothesis, WT cells were cultured in the synthetic minimal medium initially containing 2% glucose, either without a PE (i.e. cells were subjected to ethanol-mock treatment) or with the following additions: 1) PE4, PE5, PE6, PE8, PE12 or PE21 alone (each being used at the final concentration of 0.1%, 0.3%, 0.5% or 1.0%, see below); or 2) a mixture of 50 μM, 100 μM, 200 μM or 500 μM spermidine with PE4, PE5, PE6, PE8, PE12 or PE21 (each being used at the final concentration of 0.1%, 0.3%, 0.5% or 1.0%).

**Figure 9:**
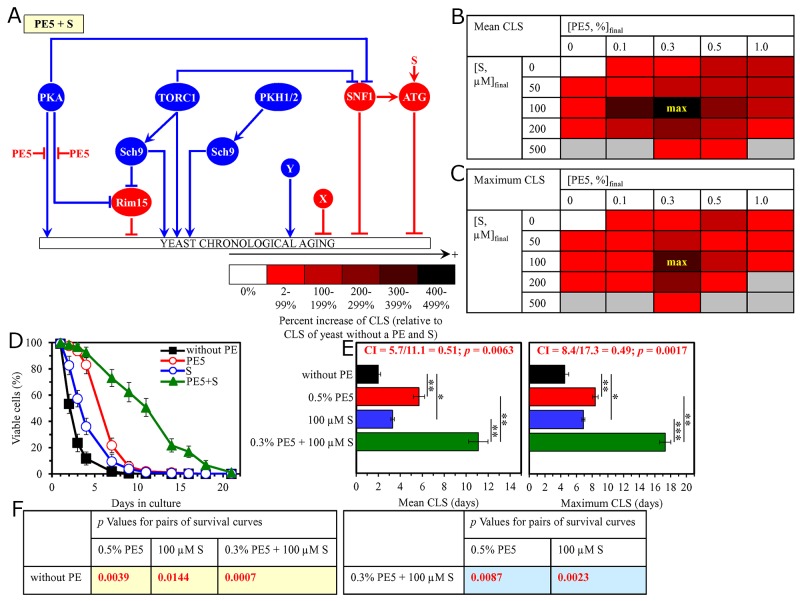
The longevity-extending efficiency of a mixture of 0.3% PE5 and 100 μM spermidine (S) statistically significantly exceeds those of PE5 and S, which were used at the optimal concentration of 0.5% or 100 μM (respectively) Thus, PE5 and S enhance the longevity-extending efficiency of each other. Hence, according to the HSA model, PE5 and S act in synergy to extend longevity of chronologically aging yeast. **(A)** PE5 and S are known to regulate different nodes of the signaling network that controls the rate of yeast chronological aging. PE5 mitigates two different branches of the pro-aging PKA pathway, whereas S stimulates the anti-aging ATG pathway. **(B, C)** WT cells were grown as described in the legend to Figure 1, with PE5 (at the final concentration of 0.1%, 0.3%, 0.5% or 1.0%) and/or S (at the final concentration of 50 μM, 100 μM, 200 μM or 500 μM), or without a PE and S. Effects of different concentrations of PE5 and S (added alone or in pairwise combinations) on the mean (B) or maximum (C) CLS of WT cells are shown. The table cell at the intersection of the column for 0.3% PE5 and the row for 100 μM S is marked ″max″ for the reason described in the legend to Figure 1. **(D, E)** WT cells were cultured as described in the legend to Figure 1, with one of the following supplements: 0.5% PE5, 100 μM S, or a mixture of 0.3% PE5 and 100 μM S. Ethanol was used as a vehicle or for mock treatment as described in the legend to Figure 1. Survival curves (D) and the mean and maximum lifespans (E) of chronologically aging WT cells cultured without a PE and S (cells were subjected to ethanol-mock treatment), with 0.5% PE5, with 100 μM S, or with the mixture of 0.3% PE5 and 100 μM S are shown. Data in D and E are presented as means ± SEM (n = 3; ^*^p < 0.05; ^**^p < 0.01; ^***^ < 0.001). The CI and *p* values in E were calculated as described in the legend to Figure 1. Data for mock-treated WT cells are replicated in graphs D and E of Figures 1–8, Figures 10–14 and Supplementary Figures 2-14. Data for WT cells cultured with 0.5% PE5 are replicated in graphs D and E of Figure 1, Figure 5, Figure 6, Supplementary Figure 3 and Supplementary Figure 4. Data for WT cells cultured with 100 μM S are replicated in graphs D and E of Figure 10 and Supplementary Figures 9-12. **(F)** *p* Values for different pairs of survival curves of WT cells cultured in the presence of 0.5% PE5, 100 μM S, a mixture of 0.3% PE5 and 100 μM S, or in the absence of a PE and S (cells were subjected to ethanol-mock treatment) are shown. Survival curves shown in (D) were compared. The *p* values are displayed on a yellow or blue color background for the reasons described in the legend to Figure 1. Abbreviations: as in the legend to Figure 1.

**Figure 10:**
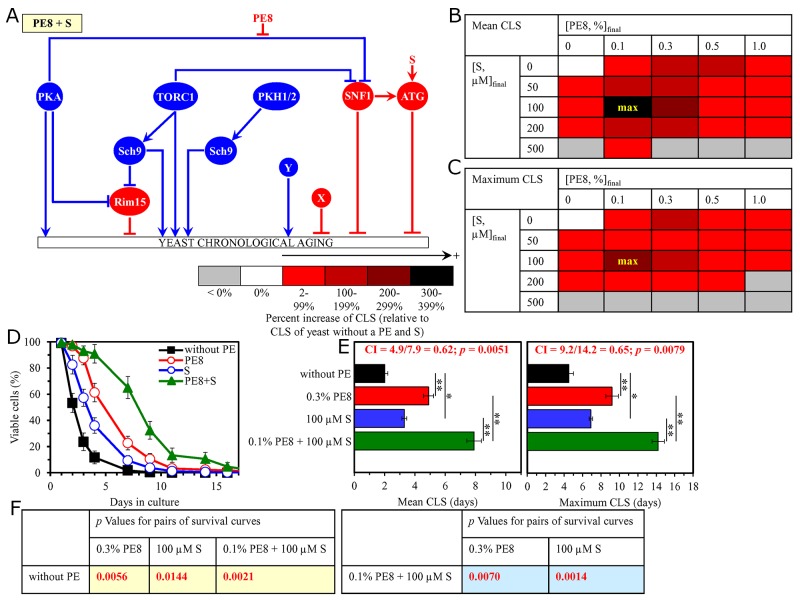
The longevity-extending efficiency of a mixture of 0.1% PE8 and 100 μM spermidine (S) statistically significantly exceeds those of PE8 and S, which were used at the optimal concentration of 0.3% or 100 μM (respectively) Thus, PE8 and S enhance the longevity-extending efficiency of each other. Hence, according to the HSA model, PE8 and S act in synergy to extend longevity of chronologically aging yeast. **(A)** PE8 and S are known to regulate different nodes of the signaling network that controls the rate of yeast chronological aging. PE8 weakens the restraining action of the pro-aging PKA pathway on the anti-aging SNF1 pathway, whereas S stimulates the anti-aging ATG pathway. **(B, C)** WT cells were grown as described in the legend to Figure 1, with PE8 (at the final concentration of 0.1%, 0.3%, 0.5% or 1.0%) and/or S (at the final concentration of 50 μM, 100 μM, 200 μM or 500 μM), or without a PE and S. Effects of different concentrations of PE8 and S (added alone or in pairwise combinations) on the mean (B) or maximum (C) CLS of WT cells are shown. The table cell at the intersection of the column for 0.1% PE8 and the row for 100 μM S is marked ″max″ for the reason described in the legend to Figure 1. **(D, E)** WT cells were cultured as described in the legend to Figure 1, with one of the following supplements: 0.3% PE8, 100 μM S, or a mixture of 0.1% PE8 and 100 μM S. Ethanol was used as a vehicle or for mock treatment as described in the legend to Figure 1. Survival curves (D) and the mean and maximum lifespans (E) of chronologically aging WT cells cultured without a PE and S (cells were subjected to ethanol-mock treatment), with 0.3% PE8, with 100 μM S, or with the mixture of 0.1% PE8 and 100 μM S are shown. Data in D and E are presented as means ± SEM (n = 3; ^*^p < 0.05; ^**^p < 0.01). The CI and *p* values in E were calculated as described in the legend to Figure 1. Data for mock-treated WT cells are replicated in graphs D and E of Figures 1–9, Figures 11–14 and Supplementary Figures 2-14. Data for WT cells cultured with 0.3% PE8 are replicated in graphs D and E of Figure 2, Figure 5, Figure 8, Supplementary Figure 5, Supplementary Figure 7 and Supplementary Figure 14. Data for WT cells cultured with 100 μM S are replicated in graphs D and E of Figure 9 and Supplementary Figures 9-12. **(F)** *p* Values for different pairs of survival curves of WT cells cultured in the presence of 0.3% PE8, 100 μM S, a mixture of 0.1% PE8 and 100 μM S, or in the absence of a PE and S (cells were subjected to ethanol-mock treatment) are shown. Survival curves shown in (D) were compared. The *p* values are displayed on a yellow or blue color background for the reasons described in the legend to Figure 1. Abbreviations: as in the legend to Figure 1.

We found that the longevity-extending efficiencies of the following pairwise combinations of spermidine and an aging-delaying PE statistically significantly exceed that of a PE within the pair which was considered as the HSA (i.e. if this PE was used alone at the optimal aging-delaying concentration): 1) a mixture of 0.1% PE4 and 100 μM spermidine as compared to 0.5% PE4 (which was considered as the HSA for both the mean and maximum CLS) (Supplementary Figure 9); 2) a mixture of 0.3% PE5 and 100 μM spermidine as compared to 0.5% PE5 (which was used as the HSA for both the mean and maximum CLS) (Figure 9); 3) a mixture of 0.5% PE6 and 100 μM spermidine as compared to 1.0% PE6 (which was considered as the HSA for both the mean and maximum CLS) (Supplementary Figure 10); 4) a mixture of 0.1% PE8 and 100 μM spermidine as compared to 0.3% PE8 (which was used as the HSA for both the mean and maximum CLS) (Figure 10); 5) a mixture of 0.1% PE12 and 100 μM spermidine as compared to 0.1% PE12 (which was considered as the HSA for both the mean and maximum CLS) (Supplementary Figure 11); and 6) a mixture of 0.1% PE21 and 100 μM spermidine as compared to 0.1% PE21 (which was used as the HSA for both the mean and maximum CLS) (Supplementary Figure 12).

These findings confirm our hypothesis that pairwise combinations of spermidine with PE4, PE5, PE6, PE8, PE12 or PE21 have synergistic effects on the extent of yeast chronological aging delay.

### Mixtures of resveratrol with PE4, PE5, PE6, PE8, PE12 or PE21 synergistically slow down yeast chronological aging

Resveratrol modulates a presently unknown pro-aging or anti-aging node that may be integrated into the signaling network that controls the rate of yeast chronological aging (Supplementary Figure 1). PE4, PE5, PE8, PE12 and PE21 affect known nodes, edges and modules of this network, whereas PE6 (akin to resveratrol) regulates a currently unidentified node of the network (Supplementary Figure 1) [87]. Based on these observations, we put forward the following hypotheses: 1) mixtures of resveratrol with PE4, PE5, PE8, PE12 or PE21 may have synergistic effects on the efficiency of yeast chronological aging delay; 2) if resveratrol and PE6 target different nodes of the network, their mixture may delay yeast chronological aging in a synergistic manner; and 3) if resveratrol and PE6 target the same node of the network, resveratrol and PE6 may not act in synergy to slow down yeast chronological aging. To test these hypotheses, we cultured WT cells in the synthetic minimal medium initially containing 2% glucose, either without a PE (i.e. cells were subjected to ethanol-mock treatment) or with the following additions: 1) PE4, PE5, PE6, PE8, PE12 or PE21 alone (each being used at the final concentration of 0.1%, 0.3%, 0.5% or 1.0%, see below); or 2) a mixture of 10 μM, 20 μM, 50 μM or 100 μM resveratrol with PE4, PE5, PE6, PE8, PE12 or PE21 (each being used at the final concentration of 0.1%, 0.3%, 0.5% or 1.0%).

**Figure 11:**
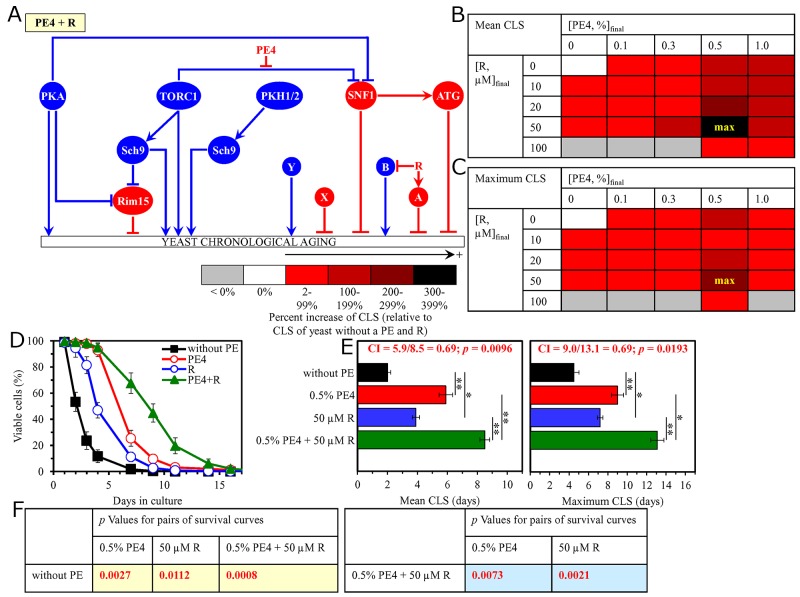
The longevity-extending efficiency of a mixture of 0.5% PE4 and 50 μM resveratrol (R) statistically significantly exceeds those of PE4 and R, which were used at the optimal concentration of 0.5% or 50 μM (respectively) Thus, PE4 and R enhance the longevity-extending efficiency of each other. Hence, according to the HSA model, PE4 and R act in synergy to extend longevity of chronologically aging yeast. **(A)** PE4 and R are known to regulate different nodes of the signaling network that controls the rate of yeast chronological aging. PE4 weakens the restraining action of the pro-aging TORC1 pathway on the anti-aging SNF1 pathway, whereas R modulates a presently unknown pro-aging or anti-aging node that may be integrated into this signaling network. **(B, C)** WT cells were grown as described in the legend to Figure 1, with PE4 (at the final concentration of 0.1%, 0.3%, 0.5% or 1.0%) and/or R (at the final concentration of 10 μM, 20 μM, 50 μM or 100 μM), or without a PE and R. Effects of different concentrations of PE4 and R (added alone or in pairwise combinations) on the mean (B) or maximum (C) CLS of WT cells are shown. The table cell at the intersection of the column for 0.5% PE4 and the row for 50 μM R is marked ″max″ for the reason described in the legend to Figure 1. **(D, E)** WT cells were cultured as described in the legend to Figure 1, with one of the following supplements: 0.5% PE4, 50 μM R, or a mixture of 0.5% PE4 and 50 μM R. Ethanol was used as a vehicle or for mock treatment as described in the legend to Figure 1. Survival curves (D) and the mean and maximum lifespans (E) of chronologically aging WT cells cultured without a PE and R (cells were subjected to ethanol-mock treatment), with 0.5% PE4, with 50 μM R, or with the mixture of 0.5% PE4 and 50 μM R are shown. Data in D and E are presented as means ± SEM (n = 3; ^*^p < 0.05; ^**^p < 0.01). The CI and *p* values in E were calculated as described in the legend to Figure 1. Data for mock-treated WT cells are replicated in graphs D and E of Figures 1–10, Figure 2, Figures 12–14 and Supplementary Figures 2-14. Data for WT cells cultured with 0.5% PE4 are replicated in graphs D and E of Figures 1–4, Figure 11, Supplementary Figure 2 and Supplementary Figure 9. Data for WT cells cultured with 50 μM R are replicated in graphs D and E of Figures 12–14, Supplementary Figure 13 and Supplementary Figure 14. **(F)** *p* Values for different pairs of survival curves of WT cells cultured in the presence of 0.5% PE4, 50 μM R, a mixture of 0.5% PE4 and 50 μM R, or in the absence of a PE and R (cells were subjected to ethanol-mock treatment) are shown. Survival curves shown in (D) were compared. The *p* values are displayed on a yellow or blue color background for the reasons described in the legend to Figure 1. Abbreviations: A, a presently unknown anti-aging node of the signaling network; B, a presently unknown pro-aging node of the signaling network; other abbreviations are as in the legend to Figure 1.

**Figure 12:**
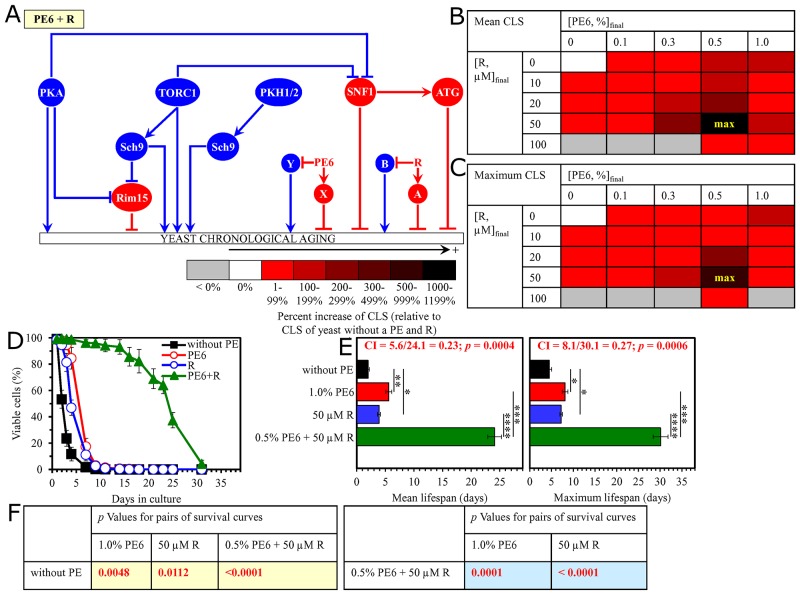
The longevity-extending efficiency of a mixture of 0.5% PE6 and 50 μM resveratrol (R) statistically significantly exceeds those of PE6 and R, which were used at the optimal concentration of 1.0% or 50 μM (respectively) Thus, PE6 and R enhance the longevity-extending efficiency of each other. Hence, according to the HSA model, PE6 and R act in synergy to extend longevity of chronologically aging yeast. **(A)** PE6 and R regulate different pro-aging or anti-aging nodes that may be integrated into the signaling network controlling the rate of yeast chronological aging; the identities of proteins that form these nodes are presently unknown. **(B, C)** WT cells were grown as described in the legend to Figure 1, with PE6 (at the final concentration of 0.1%, 0.3%, 0.5% or 1.0%) and/or R (at the final concentration of 10 μM, 20 μM, 50 μM or 100 μM), or without a PE and R. Effects of different concentrations of PE6 and R (added alone or in pairwise combinations) on the mean (B) or maximum (C) CLS of WT cells are shown. The table cell at the intersection of the column for 0.5% PE6 and the row for 50 μM R is marked ″max″ for the reason described in the legend to Figure 1. **(D, E)** WT cells were cultured as described in the legend to Figure 1, with one of the following supplements: 0.5% PE6, 50 μM R, or a mixture of 0.5% PE6 and 50 μM R. Ethanol was used as a vehicle or for mock treatment as described in the legend to Figure 1. Survival curves (D) and the mean and maximum lifespans (E) of chronologically aging WT cells cultured without a PE and R (cells were subjected to ethanol-mock treatment), with 1.0% PE6, with 50 μM R, or with the mixture of 0.5% PE6 and 50 μM R are shown. Data in D and E are presented as means ± SEM (n = 3; ^*^p < 0.05; ^**^p < 0.01; ^***^p < 0.001; ^****^p < 0.0001). The CI and *p* values in E were calculated as described in the legend to Figure 1. Data for mock-treated WT cells are replicated in graphs D and E of Figures 1–11, Figure 13, Figure 14 and Supplementary Figures 2-14. Data for WT cells cultured with 1.0% PE6 are replicated in graphs D and E of Figure 7, Supplementary Figure 2, Supplementary Figure 3, Supplementary Figure 5, Supplementary Figure 6 and Supplementary Figure 10. Data for WT cells cultured with 50 μM R are replicated in graphs D and E of Figure 11, Figure 13, Figure 14, Supplementary Figure 13 and Supplementary Figure 14. **(F)** *p* Values for different pairs of survival curves of WT cells cultured in the presence of 1.0% PE6, 50 μM R, a mixture of 0.5% PE6 and 50 μM R, or in the absence of a PE and R (cells were subjected to ethanol-mock treatment) are shown. Survival curves shown in (D) were compared. The *p* values are displayed on a yellow or blue color background for the reasons described in the legend to Figure 1. Abbreviations: as in the legend to Figure 1.

**Figure 13:**
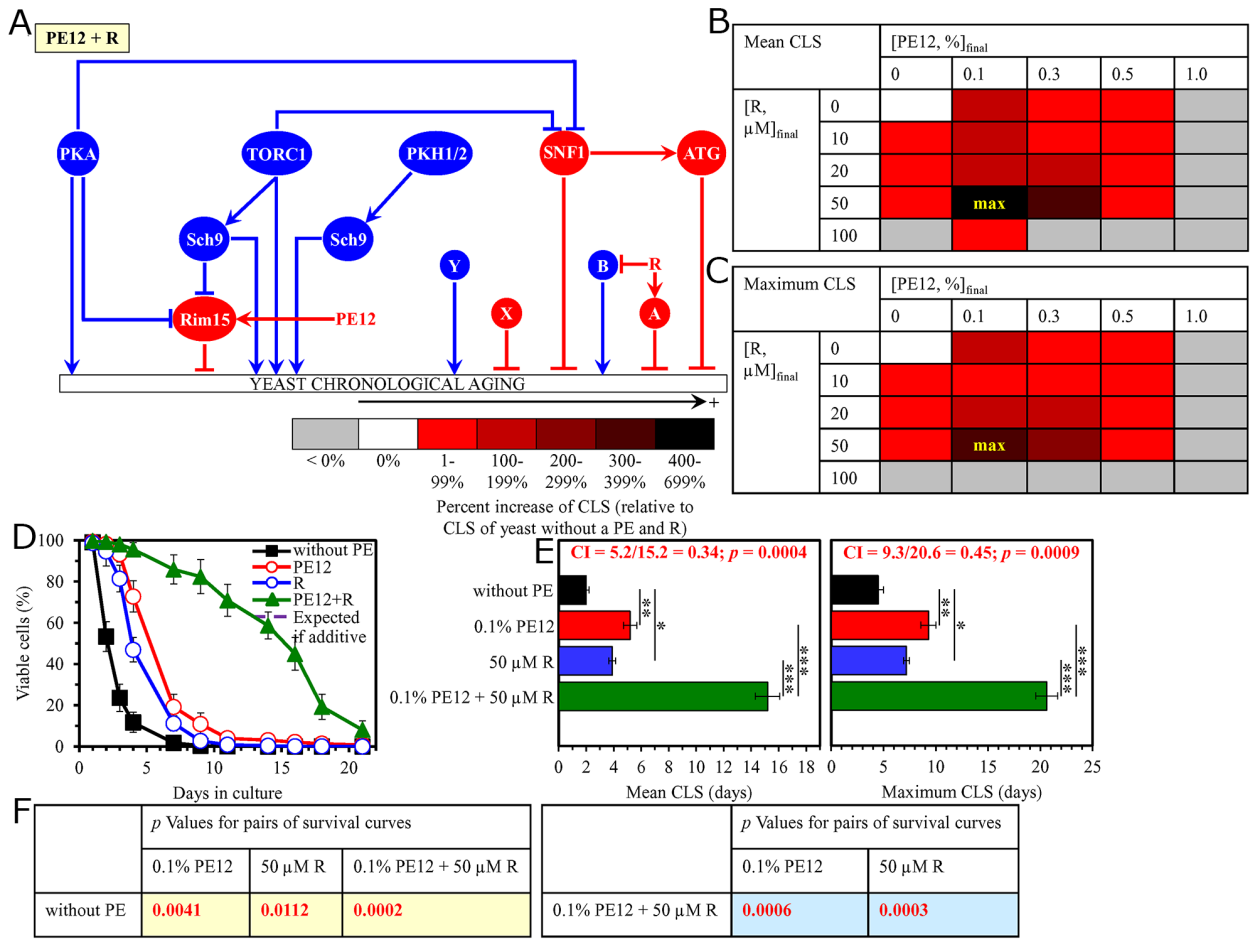
The longevity-extending efficiency of a mixture of 0.1% PE12 and 50 μM resveratrol (R) statistically significantly exceeds those of PE12 and R, which were used at the optimal concentration of 0.1% or 50 μM (respectively) Thus, PE12 and R enhance the longevity-extending efficiency of each other. Hence, according to the HSA model, PE12 and R act in synergy to extend longevity of chronologically aging yeast. **(A)** PE12 and R regulate different nodes of the signaling network that controls the rate of yeast chronological aging. PE12 stimulates the anti-aging protein kinase Rim15, whereas R modulates a presently unknown pro-aging or anti-aging node that may be integrated into this signaling network. **(B, C)** WT cells were grown as described in the legend to Figure 1, with PE12 (at the final concentration of 0.1%, 0.3%, 0.5% or 1.0%) and/or R (at the final concentration of 10 μM, 20 μM, 50 μM or 100 μM), or without a PE and R. Effects of different concentrations of PE12 and R (added alone or in pairwise combinations) on the mean (B) or maximum (C) CLS of WT cells are shown. The table cell at the intersection of the column for 0.1% PE12 and the row for 50 μM R is marked ″max″ for the reason described in the legend to Figure 1. **(D, E)** WT cells were cultured as described in the legend to Figure 1, with one of the following supplements: 0.1% PE12, 50 μM R, or a mixture of 0.1% PE12 and 50 μM R. Ethanol was used as a vehicle or for mock treatment as described in the legend to Figure 1. Survival curves (D) and the mean and maximum lifespans (E) of chronologically aging WT cells cultured without a PE and R (cells were subjected to ethanol-mock treatment), with 0.1% PE12, with 50 μM R, or with the mixture of 0.1% PE12 and 50 μM R are shown. Data in D and E are presented as means ± SEM (n = 3; ^*^p < 0.05; ^**^p < 0.01; ^***^p < 0.001). The CI and *p* values in E were calculated as described in the legend to Figure 1. Data for mock-treated WT cells are replicated in graphs D and E of Figures 1–12, Figure 14 and Supplementary Figures 2-14. Data for WT cells cultured with 0.1% PE12 are replicated in graphs D and E of Figure 3, Figure 7, Figure 8, Supplementary Figure 4, Supplementary Figure 8 and Supplementary Figure 11. Data for WT cells cultured with 50 μM R are replicated in graphs D and E of Figure 11, Figure 12, Figure 14, Supplementary Figure 13 and Supplementary Figure 14. **(F)** *p* Values for different pairs of survival curves of WT cells cultured in the presence of 0.1% PE12, 50 μM R, a mixture of 0.1% PE12 and 50 μM R, or in the absence of a PE and R (cells were subjected to ethanol-mock treatment) are shown. Survival curves shown in (D) were compared. The *p* values are displayed on a yellow or blue color background for the reasons described in the legend to Figure 1. Abbreviations: as in the legend to Figure 1.

**Figure 14:**
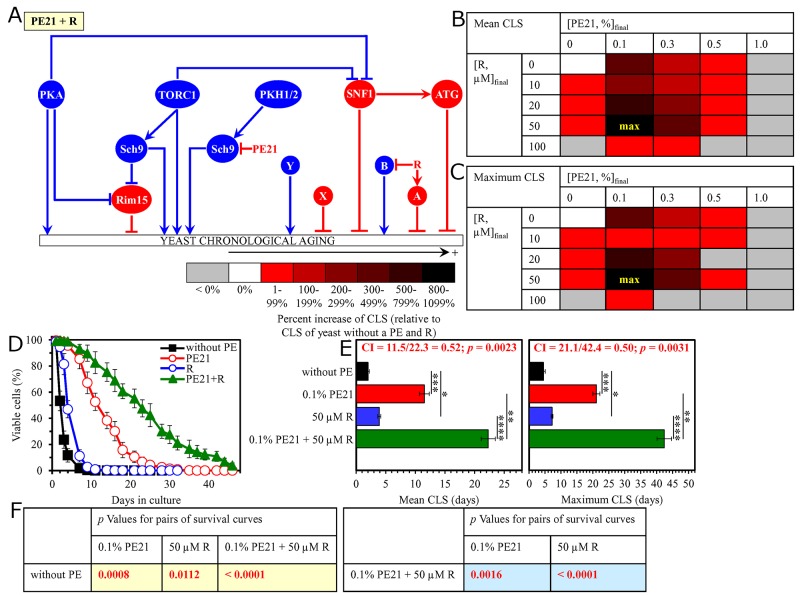
The longevity-extending efficiency of a mixture of 0.1% PE21 and 50 μM resveratrol (R) statistically significantly exceeds those of PE21 and R, which were used at the optimal concentration of 0.1% or 50 μM (respectively) Thus, PE21 and R enhance the longevity-extending efficiency of each other. Hence, according to the HSA model, PE21 and R act in synergy to extend longevity of chronologically aging yeast. **(A)** PE21 and R regulate different nodes of the signaling network that controls the rate of yeast chronological aging. PE21 mitigates a form of the pro-aging protein kinase Sch9 that is activated by the pro-aging PKH1/2 pathway, whereas R modulates a presently unknown pro-aging or anti-aging node that may be integrated into this signaling network. **(B, C)** WT cells were grown as described in the legend to Figure 1, with PE21 (at the final concentration of 0.1%, 0.3%, 0.5% or 1.0%) and/or R (at the final concentration of 10 μM, 20 μM, 50 μM or 100 μM), or without a PE and R. Effects of different concentrations of PE21 and R (added alone or in pairwise combinations) on the mean (B) or maximum (C) CLS of WT cells are shown. The table cell at the intersection of the column for 0.1% PE21 and the row for 50 μM R is marked ″max″ for the reason described in the legend to Figure 1. **(D, E)** WT cells were cultured in the synthetic minimal YNB medium initially containing 2% glucose and one of the following supplements: 0.1% PE21, 50 μM R, or a mixture of 0.1% PE21 and 50 μM R. Ethanol was used as a vehicle or for mock treatment as described in the legend to Figure 1. Survival curves (D) and the mean and maximum lifespans (E) of chronologically aging WT cells cultured without a PE and R (cells were subjected to ethanol-mock treatment), with 0.1% PE21, with 50 μM R, or with the mixture of 0.1% PE21 and 50 μM R are shown. Data in D and E are presented as means ± SEM (n = 3; ^*^p < 0.05; ^**^p < 0.01; ^***^p < 0.001; ^****^p < 0.0001). The CI and *p* values in E were calculated as described in the legend to Figure 1. Data for mock-treated WT cells are replicated in graphs D and E of Figures 1–13 and Supplementary Figures 2-14. Data for WT cells cultured with 0.1% PE12 are replicated in graphs D and E of Figure 3, Figure 7, Figure 8, Supplementary Figure 4, Supplementary Figure 8 and Supplementary Figure 11. Data for WT cells cultured with 50 μM R are replicated in graphs D and E of Figures 11–13, Supplementary Figure 13 and Supplementary Figure 14. **(F)** *p* Values for different pairs of survival curves of WT cells cultured in the presence of 0.1% PE21, 50 μM R, a mixture of 0.1% PE21 and 50 μM R, or in the absence of a PE and R (cells were subjected to ethanol-mock treatment) are shown. Survival curves shown in (D) were compared. The *p* values are displayed on a yellow or blue color background for the reasons described in the legend to Figure 1. Abbreviations: as in the legend to Figure 1.

We found that the longevity-extending efficiencies of the following mixtures of resveratrol and an aging-delaying PE are statistically significantly greater than that of a PE within the mixture which was used as the HSA (i.e. if this PE was added alone at the optimal aging-delaying concentration): 1) a mixture of 0.5% PE4 and 50 μM resveratrol as compared to 0.5% PE4 (which was used as the HSA for both the mean and maximum CLS) (Figure 11); 2) a mixture of 0.3% PE5 and 50 μM resveratrol as compared to 0.5% PE5 (which was considered as the HSA for both the mean and maximum CLS) (Supplementary Figure 13); 3) a mixture of 0.5% PE6 and 50 μM resveratrol as compared to 1.0% PE6 (which was used as the HSA for both the mean and maximum CLS) (Figure 12); 4) a mixture of 0.3% PE8 and 50 μM resveratrol as compared to 0.3% PE8 (which was considered as the HSA for both the mean and maximum CLS) (Supplementary Figure 14); 5) a mixture of 0.1% PE12 and 50 μM resveratrol as compared to 0.1% PE12 (which was used as the HSA for both the mean and maximum CLS) (Figure 13); and 6) a mixture of 0.1% PE21 and 50 μM resveratrol as compared to 0.1% PE21 (which was considered as the HSA for both the mean and maximum CLS) (Figure 14).

In sum, these findings confirm the following hypotheses: 1) mixtures of resveratrol with PE4, PE5, PE8, PE12 or PE21 exhibit synergistic effects on the efficiency of yeast chronological aging delay; and 2) if resveratrol and PE6 target different nodes of the network, a mixture of resveratrol and PE6 delays yeast chronological aging in a synergistic manner.

## DISCUSSION

The objective of this proof-of-concept study was to test our hypothesis that a mixture of two aging-delaying PEs or a combination of one of these PEs and spermidine or resveratrol may delay yeast chronological aging and extend yeast longevity in a synergistic fashion only if each of the two components of this mixture affects a different node, edge or module of the signaling network of longevity regulation. To attain this objective, we performed a systematic assessment of longevity-extending proficiencies of all possible pairwise combinations of PE4, PE5, PE6, PE8, PE12 and PE21 or of one of these PEs and spermidine or resveratrol in chronologically aging *S. cerevisiae*. In support of our hypothesis, we provided evidence that pairwise combinations of naturally-occurring chemical compounds that slow yeast chronological aging through different nodes, edges and modules of this evolutionarily conserved network exhibit synergistic effects on the magnitude of aging delay. It needs to be emphasized that studies in mice, fruit flies, aquatic invertebrates, nematodes, and budding and fission yeast have recently demonstrated that a two- or three-component combination of the aging-delaying chemical compounds that target different aging-associated processes or signaling pathways synergistically delay aging and prolong healthy lifespan [79–85]. Given that the major aspects and basic mechanisms of aging and aging-associated pathology have been conserved over the course of evolution [64, 67, 88, 91, 94–102], findings in budding yeast presented here and the above findings in other model eukaryotic organisms [79–85] support the proposed idea [74–78] that multicomponent combinations of chemical compounds that target different aging-associated processes or signaling pathways can be used for therapeutic multiplexing of aging delay and healthspan improvement in humans.

Of note, our recent study has revealed that PE4, PE5, PE6, PE8, PE12 and PE21 are geroprotectors that delay the onset and decrease the rate of yeast chronological aging by triggering a hormetic stress response and differently altering the following longevity-defining cellular processes: 1) the maintenance of mitochondrial respiration and membrane potential; 2) the preservation of reactive oxygen species homeostasis; 3) the protection of cellular proteins, membrane lipids, and mitochondrial and nuclear genomes from oxidative damage; 4) cell defense from acute oxidative and thermal stresses; and 5) the lipolytic degradation of neutral lipids deposited in lipid droplets [86]. In the future, it would be interesting to investigate how the two-component mixes of the six aging-delaying PEs that synergistically delay yeast chronological aging influence each of these cellular processes. This will allow us to gain insight into the mechanisms through which each of these pairwise combinations of PEs can delay the onset and decelerate the progression of the cellular aging process.

## MATERIALS AND METHODS

### Yeast strains, media and growth conditions

The wild-type strain *Saccharomyces cerevisiae* BY4742 (*MAT*a *his3D1 leu2D0 lys2D0 ura3D0*) and single-gene-deletion mutant strains in the BY4742 genetic background (all from Thermo Scientific/Open Biosystems) were grown in a synthetic minimal YNB medium (0.67% (w/v) Yeast Nitrogen Base without amino acids) initially containing 2% (w/v) glucose and supplemented with 20 mg/l histidine, 30 mg/l leucine, 30 mg/l lysine and 20 mg/l uracil. Cells were cultured at 30°C with rotational shaking at 200 rpm in Erlenmeyer flasks at a “flask volume/medium volume” ratio of 5:1.

### Aging-delaying PEs

PE4 (an extract from the root and rhizome of *Cimicifuga racemosa*), PE5 (an extract from the root of *Valeriana officinalis L.*), PE6 (an extract from the whole plant of *Passiflora incarnate L.*), PE8 (an extract from the leaf of *Ginkgo biloba*), PE12 (an extract from the seed of *Apium graveolens L.*) and PE21 (an extract from the bark of *Salix alba*) were used at the final concentration of 0.1% (w/v), 0.3% (w/v), 0.5% (w/v) or 1.0% (w/v) [86]. A stock solution of each PE in ethanol was made on the day of adding this PE to cell cultures. For each PE, the stock solution was added to growth medium with 2% (w/v) glucose immediately following cell inoculation into the medium. In a culture supplemented with a PE, ethanol was used as a vehicle at the final concentration of 2.5% (v/v). In the same experiment, yeast cells were also subjected to ethanol-mock treatment by being cultured in growth medium initially containing 2% glucose and 2.5% (v/v) ethanol.

### CLS assay

A sample of cells was taken from a culture at a certain day following cell inoculation and PE addition into the medium. A fraction of the sample was diluted to determine the total number of cells using a hemacytometer. Another fraction of the cell sample was diluted, and serial dilutions of cells were plated in duplicate onto YEP (1% (w/v) yeast extract, 2% (w/v) peptone) plates containing 2% (w/v) glucose as carbon source. After 2 d of incubation at 30°C, the number of colony forming units (CFU) per plate was counted. The number of CFU was defined as the number of viable cells in a sample. For each culture, the percentage of viable cells was calculated as follows: (number of viable cells per ml/total number of cells per ml) × 100. The percentage of viable cells in mid-logarithmic growth phase was set at 100%.

### Statistical analysis

Statistical analysis was performed using Microsoft Excel’s (2010) Analysis ToolPack-VBA. All data on cell survival are presented as mean ± SEM. The *p* values for comparing the means of two groups using an unpaired two-tailed *t* test were calculated with the help of the GraphPad Prism 7 statistics software. The logrank test for comparing each pair of survival curves was performed with GraphPad Prism 7. Two survival curves were considered statistically different if the *p* value was less than 0.05.

## SUPPLEMENTARY MATERIALS FIGURES AND TABLE



## Abbreviations

ATG: autophagy
AMPK: AMP-activated protein kinase
CFU: colony forming units
CI: combination index
CLS: chronological lifespan
CR: caloric restriction
HSA: highest single agent
IGF-1: insulin-like growth factor 1
PDTC: pyrrolidine dithiocarbamates
PEs: plant extracts
PKA: protein kinase A
PKH1/2: Pkb-activating kinase homologs 1 and 2
TCM: traditional Chinese medicine
Rim15: an anti-aging protein kinase
Sch9: a pro-aging protein kinase
SNF1: sucrose non-fermenting protein 1
TOR: target of rapamycin
TORC1: target of rapamycin complex 1
