# Supplementary material for: Pairwise combinations of chemical compounds that delay yeast chronological aging through different signaling pathways display synergistic effects on the extent of aging delay
Source: Oncotarget. 2019 Jan 8;10(3):313–38. doi: 10.18632/oncotarget.26553 (PMC6349451; doi:10.18632/oncotarget.26553)
Supplement: Supplementary file 1 [file oncotarget-10-313-s001.pdf]

Pairwise combinations of chemical compounds that delay yeast chronological aging through different signaling pathways display synergistic effects on the extent of aging delay

SUPPLEMENTARY MATERIALS

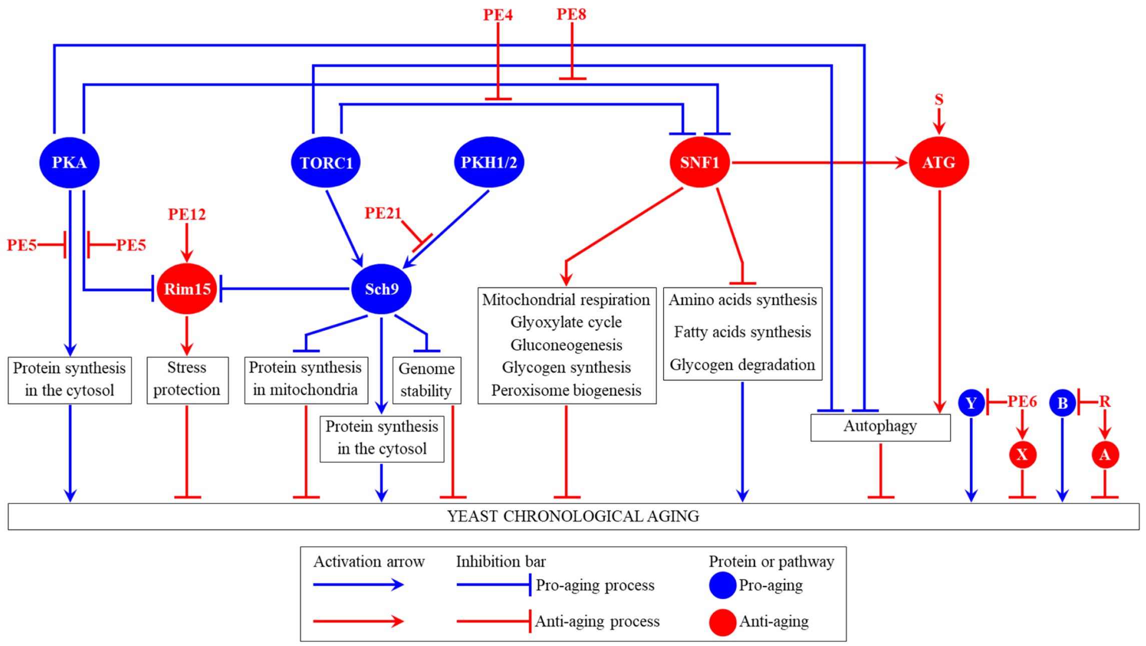

Supplementary Figure 1: PE4, PE5, PE8, PE12, PE21 and spermidine (S) delay yeast chronological aging because they regulate various pro-aging or anti-aging nodes, edges and modules of an evolutionarily conserved signaling network known to control the rate of aging. PE6 and resveratrol (R) delay yeast chronological aging by regulating a presently unknown pro-aging or anti-aging node that may be integrated into this signaling network. The signaling network that controls the rate of yeast chronological aging coordinates various longevity-defining cellular processes; these processes are named in boxes. Activation arrows and inhibition bars denote pro-aging processes (displayed in blue color) or anti-aging processes (displayed in red color). Pro-aging or anti-aging nodes (i.e. the key protein components of pro-aging or anti-aging signaling pathways) integrated into this signaling network are displayed in blue or red color, respectively. Please see text for additional details. Abbreviations: ATG, autophagy; PKA, protein kinase A; PKH1/2, Pkb-activating kinase homologs 1 and 2; Rim15, an anti-aging protein kinase; Sch9, a pro-aging protein kinase; SNF1, sucrose non-fermenting protein 1; TORC1, target of rapamycin complex 1; A and X, presently unknown anti-aging nodes of this signaling network; B and Y, presently unknown pro-aging nodes of this signaling network.

Supplementary Table 1: This study assessed how each possible pairwise combination of PE4, PE5, PE6, PE8, PE12 and PE21 or of one of these PEs and spermidine (S) or resveratrol (R) influences yeast chronological aging

	PE5	PE6	PE8	PE12	PE21	S	R
PE4	PE4 + PE5	PE4 + PE6	PE4 + PE8	PE4 + PE12	PE4 + PE21	PE4 + S	PE4 + R
PE5		PE5 + PE6	PE5 + PE8	PE5 + PE12	PE5 + PE21	PE5 + S	PE5 + R
PE6			PE6 + PE8	PE6 + PE12	PE6 + PE21	PE6 + S	PE6 + R
PE8				PE8 + PE12	PE8 + PE21	PE8 + S	PE8 + R
PE12					PE12 + PE21	PE12 + S	PE12 + R
PE21						PE21 + S	PE21 + R

These pairwise combinations are displayed on a yellow color background.

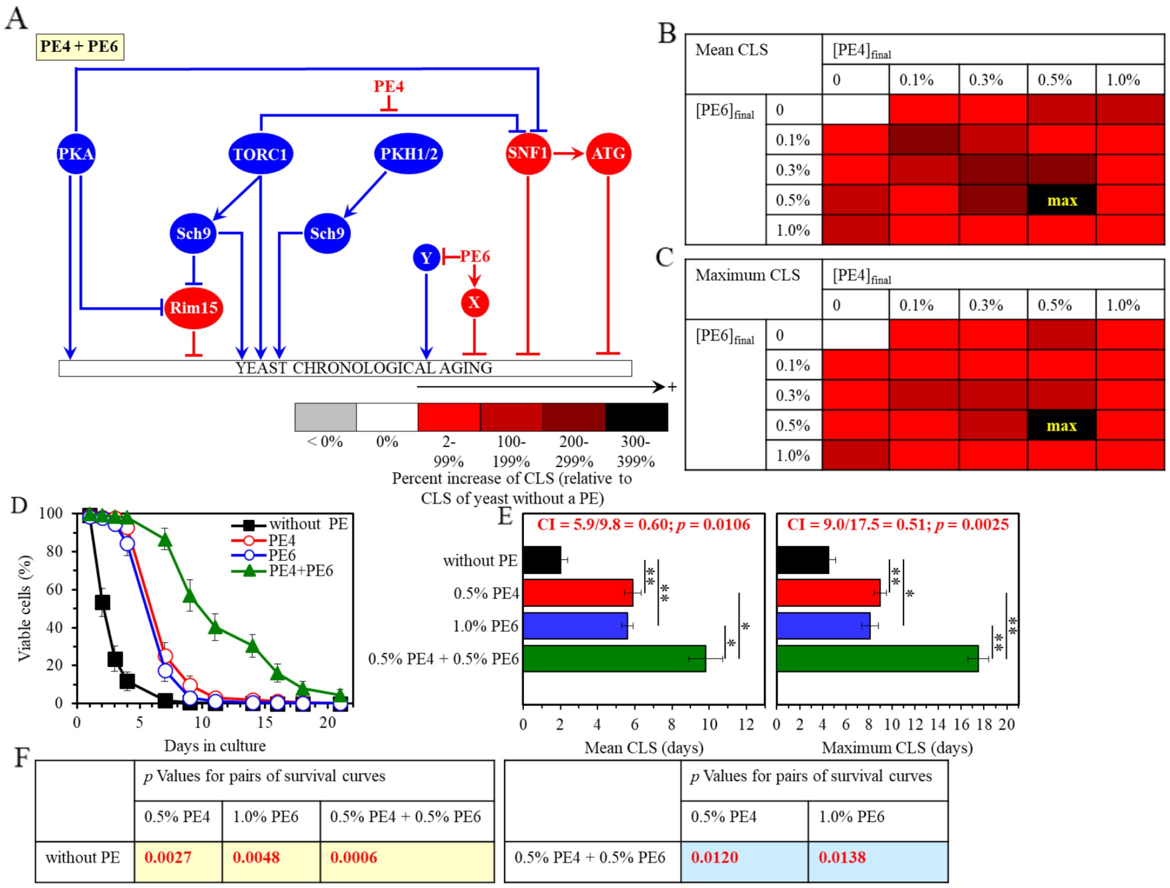

F

	p Values for pairs of survival curves		
	0.5% PE4	1.0% PE6	0.5% PE4 + 0.5% PE6
without PE	0.0027	0.0048	0.0006

	p Values for pairs of survival curves	
	0.5% PE4	1.0% PE6
0.5% PE4 + 0.5% PE6	0.0120	0.0138

Supplementary Figure 2: The longevity-extending efficiency of a mixture of 0.5% PE4 and 0.5% PE6 statistically significantly exceeds those of PE4 and PE6, which were used at the optimal concentration of 0.5% or 1.0% (respectively). Thus, PE4 and PE6 enhance the longevity-extending efficiency of each other. Hence, according to the highest single agent (HSA) model, PE4 and PE6 act in synergy to extend longevity of chronologically aging yeast. **(A)** PE4 and PE6 are known to regulate different nodes of the signaling network that controls the rate of yeast chronological aging. PE4 weakens the restraining action of the pro-aging TORC1 pathway on the anti-aging SNF1 pathway, whereas PE6 modulates a presently unknown pro-aging or anti-aging node that may be integrated into this signaling network. **(B, C)** WT cells were grown in the synthetic minimal YNB medium initially containing 2% glucose, with PE4 and/or PE6 (at the final concentration of 0.1%, 0.3%, 0.5% or 1.0%) or without a PE. Effects of different concentrations of PE4 and PE6 (added alone or in pairwise combinations) on the mean (B) or maximum (C) CLS of WT cells are shown. The table cell at the intersection of the column for 0.5% PE4 and the row for 0.5% PE6 is marked ″max″ because the mixture of 0.5% PE4 and 0.5% PE6 exhibits the highest extending effect on the mean and maximum lifespans of chronologically aging WT cells. **(D, E)** WT cells were cultured in the synthetic minimal YNB medium initially containing 2% glucose and one of the following supplements: 0.5% PE4, 1.0% PE6, or a mixture of 0.5% PE4 and 0.5% PE6. In the cultures supplemented with PE4 and/or PE6, ethanol was used as a vehicle at the final concentration of 2.5%. In the same experiment, WT cells were also subjected to ethanol-mock treatment by being cultured in the synthetic minimal YNB medium initially containing 2% glucose and 2.5% ethanol. Survival curves (D) and the mean and maximum lifespans (E) of chronologically aging WT cells cultured without a PE (cells were subjected to ethanol-mock treatment), with 0.5% PE4, with 1.0% PE6, or with the mixture of 0.5% PE4 and 0.5% PE6 are shown. Data in D and E are presented as means ± SEM (n = 3; *p < 0.05; **p < 0.01). The CI values in E were calculated as follows: CI = $CLS_{PE4}/CLS_{PE4+PE6}$ for both the mean and maximum CLS; the significance of a synergistic effect (i.e. CI < 1) is provided as the *p* value of the two-tailed *t* test for comparing the effect of a PE combination (i.e. $CLS_{PE4+PE6}$) to that of the HSA (i.e. CLS_{PE4} for both the mean and maximum CLS). Data for mock-treated WT cells are replicated in graphs D and E of Figures 1-14 and Supplementary Figures 3-14. Data for WT cells cultured with 0.5% PE4 are replicated in graphs D and E of Figures 1-4, Figure 11 and Supplementary Figure 9. Data for WT cells cultured with 1.0% PE6 are replicated in graphs D and E of Figure 7, Figure 12, Supplementary Figure 3, Supplementary Figure 5, Supplementary Figure 6 and Supplementary Figure 10. **(F)** *p* Values for different pairs of survival curves of WT cells cultured in the presence of 0.5% PE4, 1.0% PE6, a mixture of 0.5% PE4 and 0.5% PE6, or in the absence of a PE (cells were subjected to ethanol-mock treatment) are shown. Survival curves shown in (D) were compared. Two survival curves were considered statistically different if the *p* value was less than 0.05. The *p* values for comparing pairs of survival curves using the logrank test were calculated as described in Materials and Methods. The *p* values displayed on a yellow color background indicate that 0.5% PE4, 1.0% PE6, and the mixture of 0.5% PE4 and 0.5% PE6 significantly extend the CLS of WT cells. The *p* values displayed on a blue color background indicate that the CLS-extending efficiency of the mixture of 0.5% PE4 and 0.5% PE6 significantly exceeds that of 0.5% PE4 or 1.0% PE6. Abbreviations: as in the legend to Figure 1.

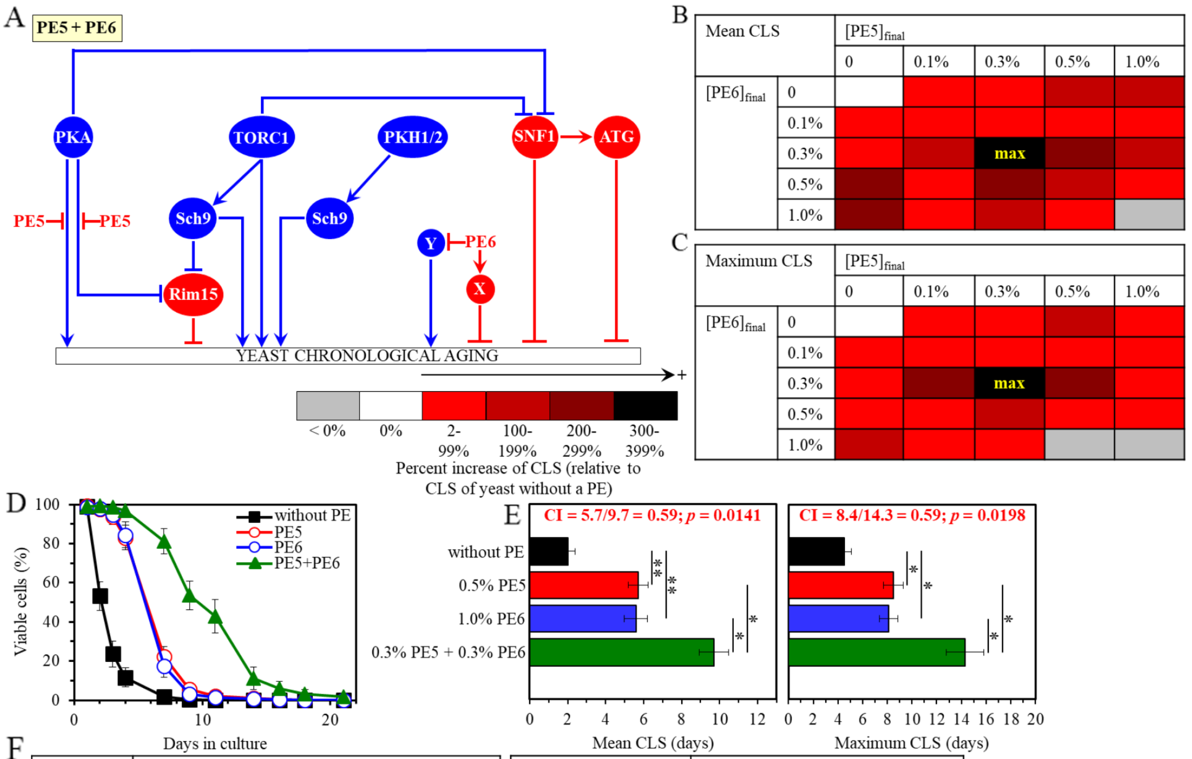

F

	p Values for pairs of survival curves		
	0.5% PE5	1.0% PE6	0.3% PE5 + 0.3% PE6
without PE	**0.0039**	**0.0048**	**0.0009**

	p Values for pairs of survival curves	
	0.5% PE5	1.0% PE6
0.3% PE5 + 0.3% PE6	**0.0125**	**0.0131**

Supplementary Figure 3: The longevity-extending efficiency of a mixture of 0.3% PE5 and 0.3% PE6 statistically significantly exceeds those of PE5 and PE6, which were used at the optimal concentration of 0.5% or 1.0% (respectively). Thus, PE5 and PE6 enhance the longevity-extending efficiency of each other. Hence, according to the highest single agent (HSA) model, PE5 and PE6 act in synergy to extend longevity of chronologically aging yeast. (A) PE5 and PE6 are known to regulate different nodes of the signaling network that controls the rate of yeast chronological aging. PE5 mitigates two different branches of the pro-aging PKA pathway, whereas PE6 modulates a presently unknown pro-aging or anti-aging node that may be integrated into this signaling network. **(B, C)** WT cells were grown in the synthetic minimal YNB medium initially containing 2% glucose, with PE5 and/or PE6 (at the final concentration of 0.1%, 0.3%, 0.5% or 1.0%) or without a PE. Effects of different concentrations of PE5 and PE6 (added alone or in pairwise combinations) on the mean (B) or maximum (C) CLS of WT cells are shown. The table cell at the intersection of the column for 0.3% PE5 and the row for 0.3% PE6 is marked "max" because the mixture of 0.3% PE5 and 0.3% PE6 exhibits the highest extending effect on the mean and maximum lifespans of chronologically aging WT cells. **(D, E)** WT cells were cultured in the synthetic minimal YNB medium initially containing 2% glucose and one of the following supplements: 0.5% PE5, 1.0% PE6, or a mixture of 0.3% PE5 and 0.3% PE6. In the cultures supplemented with PE5 and/or PE6, ethanol was used as a vehicle at the final concentration of 2.5%. In the same experiment, WT cells were also subjected to ethanol-mock treatment by being cultured in the synthetic minimal YNB medium initially containing 2% glucose and 2.5% ethanol. Survival curves (D) and the mean and maximum lifespans (E) of chronologically aging WT cells cultured without a PE (cells were subjected to ethanol-mock treatment), with 0.5% PE5, with 1.0% PE6, or with the mixture of 0.3% PE5 and 0.3% PE6 are shown. Data in D and E are presented as means ± SEM (n = 3; *p < 0.05; **p < 0.01). The CI values in E were calculated as follows: CI = $CLS_{PE5}/CLS_{PE5+PE6}$ for both the mean and maximum CLS; the significance of a synergistic effect (i.e. CI < 1) is provided as the *p* value of the two-tailed *t* test for comparing the effect of a PE combination (i.e. $CLS_{PE5+PE6}$) to that of the HSA (i.e. CLS_{PE5} for both the mean and maximum CLS). Data for mock-treated WT cells are replicated in graphs D and E of Figures 1-14, Supplementary Figure 2 and Supplementary Figures 4-14. Data for WT cells cultured with 0.5% PE5 are replicated in graphs D and E of Figure 1, Figure 5, Figure 6, Figure 9 and Supplementary Figure 4. Data for WT cells cultured with 1.0% PE6 are replicated in graphs D and E of Figure 7, Figure 12, Supplementary Figure 2, Supplementary Figure 5, Supplementary Figure 6 and Supplementary Figure 10. **(F)** *p* Values for different pairs of survival curves of WT cells cultured in the presence of 0.5% PE5, 1.0% PE6, a mixture of 0.3% PE5 and 0.3% PE6, or in the absence of a PE (cells were subjected to ethanol-mock treatment) are shown. Survival curves shown in (D) were compared. Two survival curves were considered statistically different if the *p* value was less than 0.05. The *p* values for comparing pairs of survival curves using the logrank test were calculated as described in Materials and Methods. The *p* values displayed on a yellow color background indicate that 0.5% PE5, 1.0% PE6, and the mixture of 0.3% PE5 and 0.3% PE6 significantly extend the CLS of WT cells. The *p* values displayed on a blue color background indicate that the CLS-extending efficiency of the mixture of 0.3% PE5 and 0.3% PE6 significantly exceeds that of 0.5% PE5 or 1.0% PE6. Abbreviations: as in the legend to Figure 1.

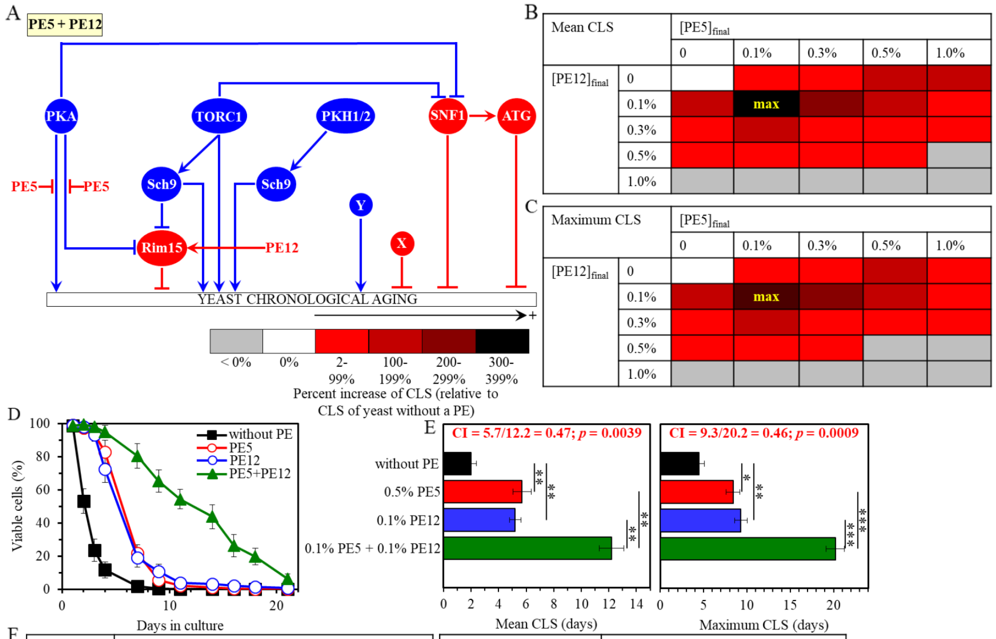

F

	p Values for pairs of survival curves		
	0.5% PE5	0.1% PE12	0.1% PE5 + 0.1% PE12
without PE	**0.0039**	**0.0041**	**0.0005**

	p Values for pairs of survival curves	
	0.5% PE5	0.1% PE12
0.1% PE5 + 0.1% PE12	**0.0032**	**0.0025**

Supplementary Figure 4: The longevity-extending efficiency of a mixture of 0.1% PE5 and 0.1% PE12 statistically significantly exceeds those of PE5 and PE12, which were used at the optimal concentration of 0.5% or 0.1% (respectively). Thus, PE5 and PE12 enhance the longevity-extending efficiency of each other. Hence, according to the highest single agent (HSA) model, PE5 and PE12 act in synergy to extend longevity of chronologically aging yeast. **(A)** PE5 and PE12 are known to regulate different nodes of the signaling network that controls the rate of yeast chronological aging. PE5 mitigates two different branches of the pro-aging PKA pathway, whereas PE12 stimulates the anti-aging protein kinase Rim15. **(B, C)** WT cells were grown in the synthetic minimal YNB medium initially containing 2% glucose, with PE5 and/or PE12 (at the final concentration of 0.1%, 0.3%, 0.5% or 1.0%) or without a PE. Effects of different concentrations of PE5 and PE12 (added alone or in pairwise combinations) on the mean (B) or maximum (C) CLS of WT cells are shown. The table cell at the intersection of the column for 0.1% PE5 and the row for 0.1% PE12 is marked ″max″ because the mixture of 0.1% PE5 and 0.1% PE12 exhibits the highest extending effect on the mean and maximum lifespans of chronologically aging WT cells. **(D, E)** WT cells were cultured in the synthetic minimal YNB medium initially containing 2% glucose and one of the following supplements: 0.5% PE5, 0.1% PE12, or a mixture of 0.1% PE5 and 0.1% PE12. In the cultures supplemented with PE5 and/or PE12, ethanol was used as a vehicle at the final concentration of 2.5%. In the same experiment, WT cells were also subjected to ethanol-mock treatment by being cultured in the synthetic minimal YNB medium initially containing 2% glucose and 2.5% ethanol. Survival curves (D) and the mean and maximum lifespans (E) of chronologically aging WT cells cultured without a PE (cells were subjected to ethanol-mock treatment), with 0.5% PE5, with 0.1% PE12, or with the mixture of 0.1% PE5 and 0.1% PE12 are shown. Data in D and E are presented as means ± SEM (n = 3; *p < 0.05; **p < 0.01; *** < 0.001). The CI values in E were calculated as follows: $CI = CLS_{PE5}/CLS_{PE5+PE12}$ for the mean CLS and $CI = CLS_{PE12}/CLS_{PE5+PE12}$ for the maximum CLS; the significance of a synergistic effect (i.e. CI < 1) is provided as the *p* value of the two-tailed *t* test for comparing the effect of a PE combination (i.e. $CLS_{PE5+PE12}$) to that of the HSA (i.e. CLS_{PE5} for the mean CLS and CLS_{PE12} for the maximum CLS). Data for mock-treated WT cells are replicated in graphs D and E of Figures 1-14, Supplementary Figure 2, Supplementary Figure 3 and Supplementary Figure 5-14. Data for WT cells cultured with 0.5% PE5 are replicated in graphs D and E of Figure 1, Figure 5, Figure 6, Figure 9 and Supplementary Figure 3. Data for WT cells cultured with 0.1% PE12 are replicated in graphs D and E of Figure 3, Figure 7, Figure 8, Figure 13, Supplementary Figure 4, Supplementary Figure 8 and Supplementary Figure 11. **(F)** *p* Values for different pairs of survival curves of WT cells cultured in the presence of 0.5% PE5, 0.1% PE12, a mixture of 0.1% PE5 and 0.1% PE12, or in the absence of a PE (cells were subjected to ethanol-mock treatment) are shown. Survival curves shown in (D) were compared. Two survival curves were considered statistically different if the *p* value was less than 0.05. The *p* values for comparing pairs of survival curves using the logrank test were calculated as described in Materials and Methods. The *p* values displayed on a yellow color background indicate that 0.5% PE5, 0.1% PE12, and the mixture of 0.1% PE5 and 0.1% PE12 significantly extend the CLS of WT cells. The *p* values displayed on a blue color background indicate that the CLS-extending efficiency of the mixture of 0.1% PE5 and 0.1% PE12 significantly exceeds that of 0.5% PE5 or 0.1% PE12. Abbreviations: as in the legend to Figure 1.

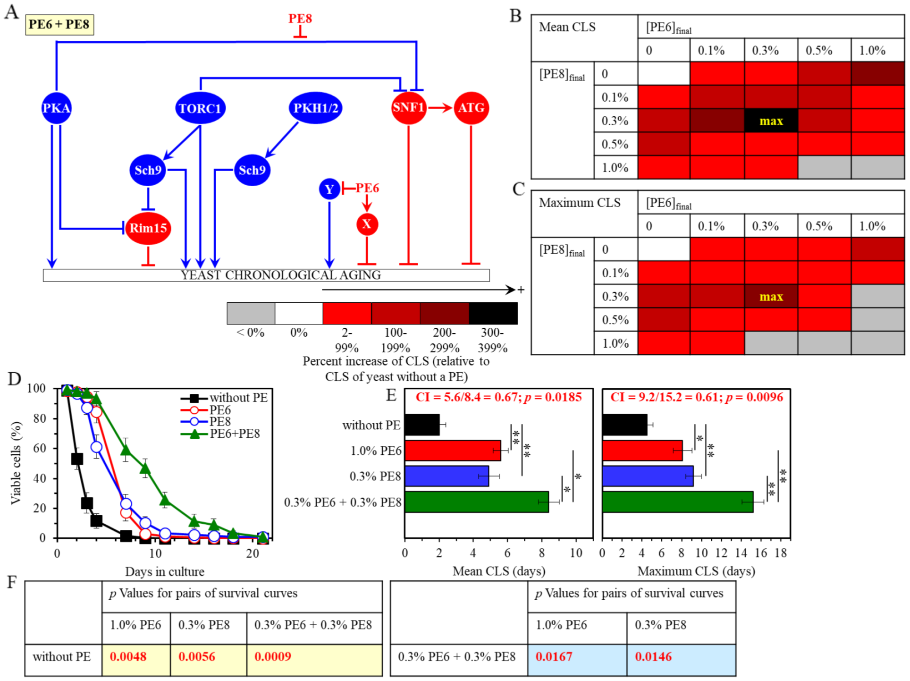

Supplementary Figure 5: The longevity-extending efficiency of a mixture of 0.3% PE6 and 0.3% PE8 statistically significantly exceeds those of PE6 and PE8, which were used at the optimal concentration of 1.0% or 0.3% (respectively). Thus, PE6 and PE8 enhance the longevity-extending efficiency of each other. Hence, according to the highest single agent (HSA) model, PE6 and PE8 act in synergy to extend longevity of chronologically aging yeast. (A) PE6 and PE8 are known to regulate different nodes of the signaling network that controls the rate of yeast chronological aging. PE8 weakens the restraining action of the pro-aging PKA pathway on the anti-aging SNF1 pathway, whereas PE6 modulates a presently unknown pro-aging or anti-aging node that may be integrated into this signaling network. **(B, C)** WT cells were grown in the synthetic minimal YNB medium initially containing 2% glucose, with PE6 and/or PE8 (at the final concentration of 0.1%, 0.3%, 0.5% or 1.0%) or without a PE. Effects of different concentrations of PE6 and PE8 (added alone or in pairwise combinations) on the mean **(B)** or maximum **(C)** CLS of WT cells are shown. The table cell at the intersection of the column for 0.3% PE6 and the row for 0.3% PE8 is marked ″max″ because the mixture of 0.3% PE6 and 0.3% PE8 exhibits the highest extending effect on the mean and maximum lifespans of chronologically aging WT cells. **(D, E)** WT cells were cultured in the synthetic minimal YNB medium initially containing 2% glucose and one of the following supplements: 1.0% PE6, 0.3% PE8, or a mixture of 0.3% PE6 and 0.3% PE8. In the cultures supplemented with PE6 and/or PE8, ethanol was used as a vehicle at the final concentration of 2.5%. In the same experiment, WT cells were also subjected to ethanol-mock treatment by being cultured in the synthetic minimal YNB medium initially containing 2% glucose and 2.5% ethanol. Survival curves (D) and the mean and maximum lifespans (E) of chronologically aging WT cells cultured without a PE (cells were subjected to ethanol-mock treatment), with 1.0% PE6, with 0.3% PE8, or with the mixture of 0.3% PE6 and 0.3% PE8 are shown. Data in D and E are presented as means ± SEM (n = 3; $*p < 0.05$; $**p < 0.01$). The CI values in E were calculated as follows: CI = $CLS_{PE6}/CLS_{PE6+PE8}$ for the mean CLS and CI = $CLS_{PE8}/CLS_{PE6+PE8}$ for the maximum CLS; the significance of a synergistic effect (i.e. CI < 1) is provided as the *p* value of the two-tailed *t* test for comparing the effect of a PE combination (i.e. $CLS_{PE6+PE8}$) to that of the HSA (i.e. CLS_{PE6} for the mean CLS and CLS_{PE8} for the maximum CLS). Data for mock-treated WT cells are replicated in graphs D and E of Figures 1-14, Supplementary Figures 2-4 and Supplementary Figures 6-14. Data for WT cells cultured with 1.0% PE6 are replicated in graphs D and E of Figure 7, Figure 12, Supplementary Figure 2, Supplementary Figure 3, Supplementary Figure 6 and Supplementary Figure 10. Data for WT cells cultured with 0.3% PE8 are replicated in graphs D and E of Figure 2, Figure 5, Figure 8, Figure 10, Supplementary Figure 7 and Supplementary Figure 14. **(F)** *p* Values for different pairs of survival curves of WT cells cultured in the presence of 1.0% PE6, 0.3% PE8, a mixture of 0.3% PE6 and 0.3% PE8, or in the absence of a PE (cells were subjected to ethanol-mock treatment) are shown. Survival curves shown in (D) were compared. Two survival curves were considered statistically different if the *p* value was less than 0.05. The *p* values for comparing pairs of survival curves using the logrank test were calculated as described in Materials and Methods. The *p* values displayed on a yellow color background indicate that 1.0% PE6, 0.3% PE8, and the mixture of 0.3% PE6 and 0.3% PE8 significantly extend the CLS of WT cells. The *p* values displayed on a blue color background indicate that the CLS-extending efficiency of the mixture of 0.3% PE6 and 0.3% PE8 significantly exceeds that of 1.0% PE6 or 0.3% PE8. Abbreviations: as in the legend to Figure 1.

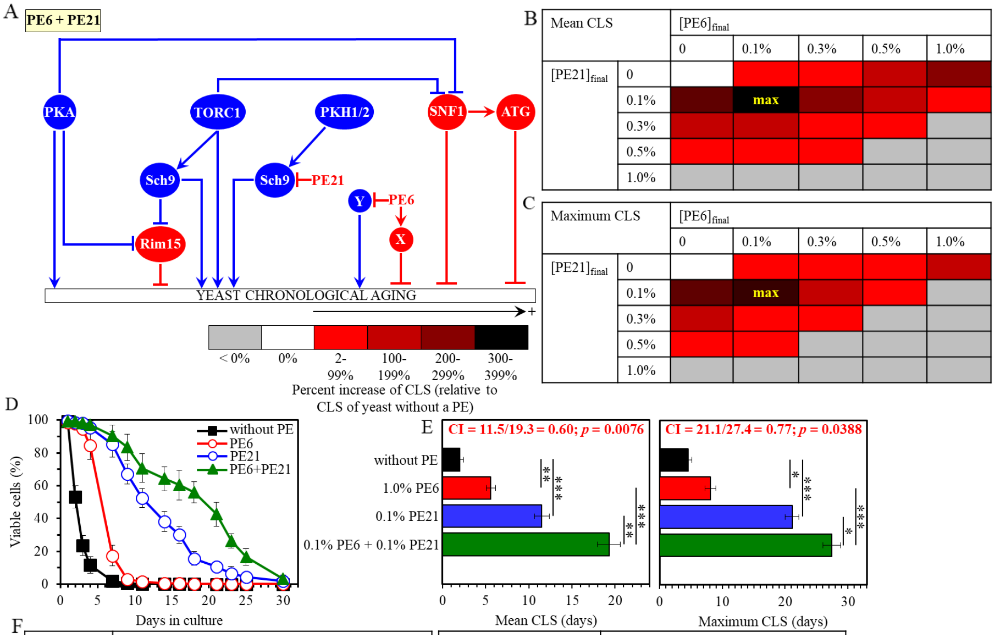

F

	p Values for pairs of survival curves		
	1.0% PE6	0.1% PE21	0.1% PE6 + 0.1% PE21
without PE	**0.0048**	**0.0008**	**0.0002**

	p Values for pairs of survival curves	
	1.0% PE6	0.1% PE21
0.1% PE6 + 0.1% PE21	**0.0011**	**0.0069**

Supplementary Figure 6: The longevity-extending efficiency of a mixture of 0.1% PE6 and 0.1% PE21 statistically significantly exceeds those of PE6 and PE21, which were used at the optimal concentration of 1.0% or 0.1% (respectively). Thus, PE6 and PE21 enhance the longevity-extending efficiency of each other. Hence, according to the highest single agent (HSA) model, PE6 and PE21 act in synergy to extend longevity of chronologically aging yeast. **(A)** PE6 and PE21 are known to regulate different nodes of the signaling network that controls the rate of yeast chronological aging. PE21 mitigates a form of the pro-aging protein kinase Sch9 that is activated by the pro-aging PKH1/2 pathway, whereas PE6 modulates a presently unknown pro-aging or anti-aging node that may be integrated into this signaling network. **(B, C)** WT cells were grown in the synthetic minimal YNB medium initially containing 2% glucose, with PE6 and/or PE21 (at the final concentration of 0.1%, 0.3%, 0.5% or 1.0%) or without a PE. Effects of different concentrations of PE6 and PE21 (added alone or in pairwise combinations) on the mean (B) or maximum (C) CLS of WT cells are shown. The table cell at the intersection of the column for 0.1% PE6 and the row for 0.1% PE21 is marked "max" because the mixture of 0.1% PE6 and 0.1% PE21 exhibits the highest extending effect on the mean and maximum lifespans of chronologically aging WT cells. **(D, E)** WT cells were cultured in the synthetic minimal YNB medium initially containing 2% glucose and one of the following supplements: 1.0% PE6, 0.1% PE21, or a mixture of 0.1% PE6 and 0.1% PE21. In the cultures supplemented with PE6 and/or PE21, ethanol was used as a vehicle at the final concentration of 2.5%. In the same experiment, WT cells were also subjected to ethanol-mock treatment by being cultured in the synthetic minimal YNB medium initially containing 2% glucose and 2.5% ethanol. Survival curves (D) and the mean and maximum lifespans (E) of chronologically aging WT cells cultured without a PE (cells were subjected to ethanol-mock treatment), with 1.0% PE6, with 0.1% PE21, or with the mixture of 0.1% PE6 and 0.1% PE21 are shown. Data in D and E are presented as means ± SEM (n = 3; *p < 0.05; **p < 0.01; *** < 0.001). The CI values in E were calculated as follows: $CI = CLS_{PE21}/CLS_{PE6+PE21}$ for both the mean and maximum CLS; the significance of a synergistic effect (i.e. CI < 1) is provided as the *p* value of the two-tailed *t* test for comparing the effect of a PE combination (i.e. $CLS_{PE6+PE21}$) to that of the HSA (i.e. CLS_{PE21} for both the mean and maximum CLS). Data for mock-treated WT cells are replicated in graphs D and E of Figures 1-14, Supplementary Figures 2-5 and Supplementary Figures 7-14. Data for WT cells cultured with 1.0% PE6 are replicated in graphs D and E of Figure 7, Figure 12, Supplementary Figure 2, Supplementary Figure 3, Supplementary Figure 5 and Supplementary Figure 10. Data for WT cells cultured with 0.1% PE21 are replicated in graphs D and E of Figure 4, Figure 6, Figure 14, Supplementary Figure 7, Supplementary Figure 8 and Supplementary Figure 12. **(F)** *p* Values for different pairs of survival curves of WT cells cultured in the presence of 1.0% PE6, 0.1% PE21, a mixture of 0.1% PE6 and 0.1% PE21, or in the absence of a PE (cells were subjected to ethanol-mock treatment) are shown. Survival curves shown in (D) were compared. Two survival curves were considered statistically different if the *p* value was less than 0.05. The *p* values for comparing pairs of survival curves using the logrank test were calculated as described in Materials and Methods. The *p* values displayed on a yellow color background indicate that 1.0% PE6, 0.1% PE21, and the mixture of 0.1% PE6 and 0.1% PE21 significantly extend the CLS of WT cells. The *p* values displayed on a blue color background indicate that the CLS-extending efficiency of the mixture of 0.1% PE6 and 0.1% PE21 significantly exceeds that of 1.0% PE6 or 0.1% PE21. Abbreviations: as in the legend to Figure 1.

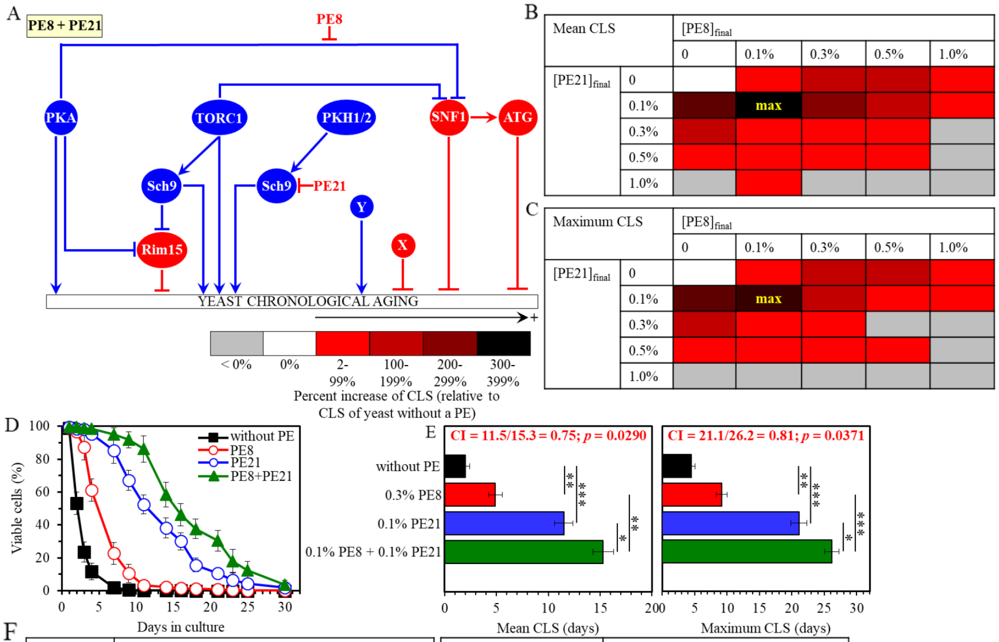

F

	p Values for pairs of survival curves		
	0.3% PE8	0.1% PE21	0.1% PE8 + 0.1% PE21
without PE	0.0056	0.0008	0.0002

	p Values for pairs of survival curves	
	0.3% PE8	0.1% PE21
0.1% PE8 + 0.1% PE21	0.0014	0.0227

Supplementary Figure 7: The longevity-extending efficiency of a mixture of 0.1% PE8 and 0.1% PE21 statistically significantly exceeds those of PE8 and PE21, which were used at the optimal concentration of 0.3% or 0.1% (respectively). Thus, PE8 and PE21 enhance the longevity-extending efficiency of each other. Hence, according to the highest single agent (HSA) model, PE8 and PE21 act in synergy to extend longevity of chronologically aging yeast. **(A)** PE8 and PE21 are known to regulate different nodes of the signaling network that controls the rate of yeast chronological aging. PE8 weakens the restraining action of the pro-aging PKA pathway on the anti-aging SNF1 pathway, whereas PE21 mitigates a form of the pro-aging protein kinase Sch9 that is activated by the pro-aging PKH1/2 pathway. **(B, C)** WT cells were grown in the synthetic minimal YNB medium initially containing 2% glucose, with PE8 and/or PE21 (at the final concentration of 0.1%, 0.3%, 0.5% or 1.0%) or without a PE. Effects of different concentrations of PE6 and PE21 (added alone or in pairwise combinations) on the mean (B) or maximum (C) CLS of WT cells are shown. The table cell at the intersection of the column for 0.1% PE8 and the row for 0.1% PE21 is marked "max" because the mixture of 0.1% PE8 and 0.1% PE21 exhibits the highest extending effect on the mean and maximum lifespans of chronologically aging WT cells. **(D, E)** WT cells were cultured in the synthetic minimal YNB medium initially containing 2% glucose and one of the following supplements: 0.3% PE8, 0.1% PE21, or a mixture of 0.1% PE8 and 0.1% PE21. In the cultures supplemented with PE8 and/or PE21, ethanol was used as a vehicle at the final concentration of 2.5%. In the same experiment, WT cells were also subjected to ethanol-mock treatment by being cultured in the synthetic minimal YNB medium initially containing 2% glucose and 2.5% ethanol. Survival curves (D) and the mean and maximum lifespans (E) of chronologically aging WT cells cultured without a PE (cells were subjected to ethanol-mock treatment), with 0.3% PE8, with 0.1% PE21, or with the mixture of 0.1% PE8 and 0.1% PE21 are shown. Data in D and E are presented as means ± SEM (n = 3; *p < 0.05; **p < 0.01; *** < 0.001). The CI values in E were calculated as follows: CI = $CLS_{PE21}/CLS_{PE8+PE21}$ for both the mean and maximum CLS; the significance of a synergistic effect (i.e. CI < 1) is provided as the *p* value of the two-tailed *t* test for comparing the effect of a PE combination (i.e. $CLS_{PE8+PE21}$) to that of the HSA (i.e. CLS_{PE21} for both the mean and maximum CLS). Data for mock-treated WT cells are replicated in graphs D and E of Figures 1-14, Supplementary Figures 2-6, Supplementary Figures 8-14. Data for WT cells cultured with 0.3% PE8 are replicated in graphs D and E of Figure 2, Figure 5, Figure 8, Figure 10, Supplementary Figure 5 and Supplementary Figure 14. Data for WT cells cultured with 0.1% PE21 are replicated in graphs D and E of Figure 4, Figure 6, Figure 14, Supplementary Figure 6, Supplementary Figure 8 and Supplementary Figure 12. **(F)** *p* Values for different pairs of survival curves of WT cells cultured in the presence of 0.3% PE8, 0.1% PE21, a mixture of 0.1% PE8 and 0.1% PE21, or in the absence of a PE (cells were subjected to ethanol-mock treatment) are shown. Survival curves shown in (D) were compared. Two survival curves were considered statistically different if the *p* value was less than 0.05. The *p* values for comparing pairs of survival curves using the logrank test were calculated as described in Materials and Methods. The *p* values displayed on a yellow color background indicate that 0.3% PE8, 0.1% PE21, and the mixture of 0.1% PE8 and 0.1% PE21 significantly extend the CLS of WT cells. The *p* values displayed on a blue color background indicate that the CLS-extending efficiency of the mixture of 0.1% PE8 and 0.1% PE21 significantly exceeds that of 0.3% PE8 or 0.1% PE21. Abbreviations: as in the legend to Figure 1.

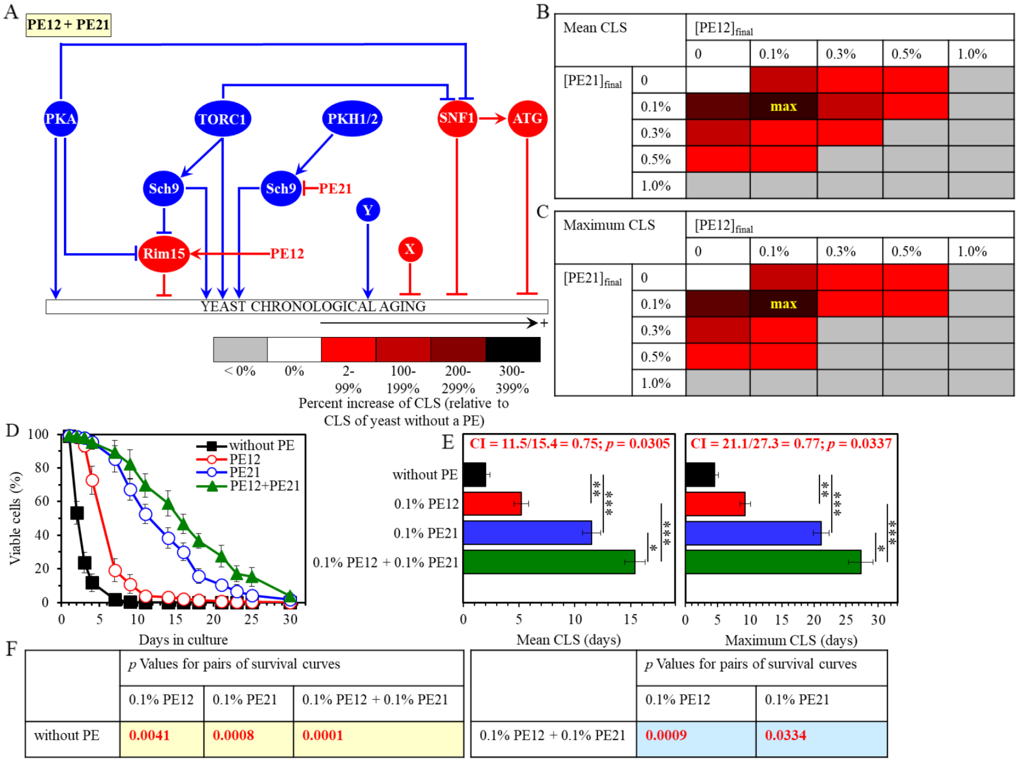

F

	p Values for pairs of survival curves		
	0.1% PE12	0.1% PE21	0.1% PE12 + 0.1% PE21
without PE	0.0041	0.0008	0.0001

	p Values for pairs of survival curves	
	0.1% PE12	0.1% PE21
0.1% PE12 + 0.1% PE21	0.0009	0.0334

Supplementary Figure 8: The longevity-extending efficiency of a mixture of 0.1% PE12 and 0.1% PE21 statistically significantly exceeds those of PE12 and PE21, each being used at the optimal concentration of 0.1%. Thus, PE12 and PE21 enhance the longevity-extending efficiency of each other. Hence, according to the highest single agent (HSA) model, PE12 and PE21 act in synergy to extend longevity of chronologically aging yeast. (A) PE12 and PE21 are known to regulate different nodes of the signaling network that controls the rate of yeast chronological aging. PE12 stimulates the anti-aging protein kinase Rim15, whereas PE21 mitigates a form of the pro-aging protein kinase Sch9 that is activated by the pro-aging PKH1/2 pathway. **(B, C)** WT cells were grown in the synthetic minimal YNB medium initially containing 2% glucose, with PE12 and/or PE21 (at the final concentration of 0.1%, 0.3%, 0.5% or 1.0%) or without a PE. Effects of different concentrations of PE12 and PE21 (added alone or in pairwise combinations) on the mean (B) or maximum (C) CLS of WT cells are shown. The table cell at the intersection of the column for 0.1% PE12 and the row for 0.1% PE21 is marked "max" because the mixture of 0.1% PE12 and 0.1% PE21 exhibits the highest extending effect on the mean and maximum lifespans of chronologically aging WT cells. **(D, E)** WT cells were cultured in the synthetic minimal YNB medium initially containing 2% glucose and one of the following supplements: 0.1% PE12, 0.1% PE21, or a mixture of 0.1% PE12 and 0.1% PE21. In the cultures supplemented with PE12 and/or PE21, ethanol was used as a vehicle at the final concentration of 2.5%. In the same experiment, WT cells were also subjected to ethanol-mock treatment by being cultured in the synthetic minimal YNB medium initially containing 2% glucose and 2.5% ethanol. Survival curves (D) and the mean and maximum lifespans (E) of chronologically aging WT cells cultured without a PE (cells were subjected to ethanol-mock treatment), with 0.1% PE12, with 0.1% PE21, or with the mixture of 0.1% PE12 and 0.1% PE21 are shown. Data in D and E are presented as means ± SEM ($n = 3$; $*p < 0.05$; $**p < 0.01$; $*** < 0.001$). The CI values in E were calculated as follows: $CI = CLS_{PE21}/CLS_{PE12+PE21}$ for both the mean and maximum CLS; the significance of a synergistic effect (i.e. $CI < 1$) is provided as the *p* value of the two-tailed *t* test for comparing the effect of a PE combination (i.e. $CLS_{PE12+PE21}$) to that of the HSA (i.e. CLS_{PE21} for both the mean and maximum CLS). Data for mock-treated WT cells are replicated in graphs D and E of Figures 1-14, Supplementary Figures 2-7 and Supplementary Figures 9-14. Data for WT cells cultured with 0.1% PE12 are replicated in graphs D and E of Figure 3, Figure 7, Figure 8, Figure 13, Supplementary Figure 4 and Supplementary Figure 11. Data for WT cells cultured with 0.1% PE21 are replicated in graphs D and E of Figure 4, Figure 6, Figure 14, Supplementary Figure 6, Supplementary Figure 7 and Supplementary Figure 12. **(F)** *p* Values for different pairs of survival curves of WT cells cultured in the presence of 0.1% PE12, 0.1% PE21, a mixture of 0.1% PE12 and 0.1% PE21, or in the absence of a PE (cells were subjected to ethanol-mock treatment) are shown. Survival curves shown in (D) were compared. Two survival curves were considered statistically different if the *p* value was less than 0.05. The *p* values for comparing pairs of survival curves using the logrank test were calculated as described in Materials and Methods. The *p* values displayed on a yellow color background indicate that 0.1% PE12, 0.1% PE21, and the mixture of 0.1% PE12 and 0.1% PE21 significantly extend the CLS of WT cells. The *p* values displayed on a blue color background indicate that the CLS-extending efficiency of the mixture of 0.1% PE12 and 0.1% PE21 significantly exceeds that of 0.1% PE12 or 0.1% PE21. Abbreviations: as in the legend to Figure 1.

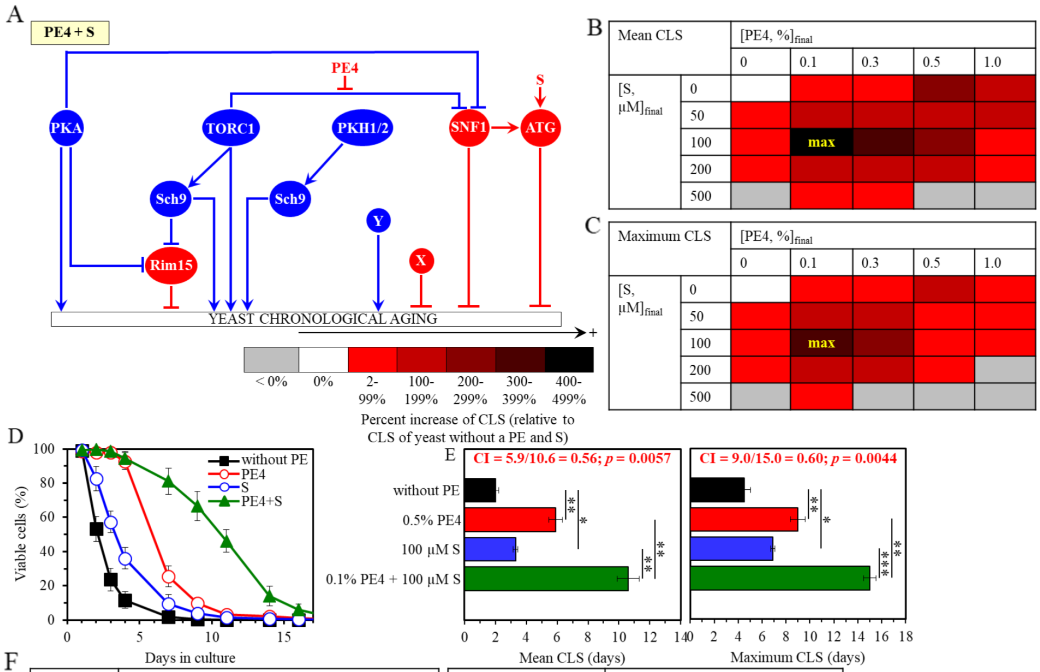

F

	p Values for pairs of survival curves		
	0.5% PE4	100 µM S	0.1% PE4 + 100 µM S
without PE	**0.0027**	**0.0144**	**0.0005**

	p Values for pairs of survival curves	
	0.5% PE4	100 µM S
0.1% PE4 + 100 µM S	**0.0048**	**0.0018**

Supplementary Figure 9: The longevity-extending efficiency of a mixture of 0.1% PE4 and 100 µM spermidine (S) statistically significantly exceeds those of PE4 and S, which were used at the optimal concentration of 0.5% or 100 µM (respectively). Thus, PE4 and S enhance the longevity-extending efficiency of each other. Hence, according to the highest single agent (HSA) model, PE4 and S act in synergy to extend longevity of chronologically aging yeast. (A) PE4 and S are known to regulate different nodes of the signaling network that controls the rate of yeast chronological aging. PE4 weakens the restraining action of the pro-aging TORC1 pathway on the anti-aging SNF1 pathway, whereas S stimulates the anti-aging ATG pathway. **(B, C)** WT cells were grown in the synthetic minimal YNB medium initially containing 2% glucose, with PE4 (at the final concentration of 0.1%, 0.3%, 0.5% or 1.0%) and/or S (at the final concentration of 50 µM, 100 µM, 200 µM or 500 µM), or without a PE and S. Effects of different concentrations of PE4 and S (added alone or in pairwise combinations) on the mean (B) or maximum (C) CLS of WT cells are shown. The table cell at the intersection of the column for 0.1% PE4 and the row for 100 µM S is marked ″max″ because the mixture of 0.1% PE4 and 100 µM S exhibits the highest extending effect on the mean and maximum lifespans of chronologically aging WT cells. **(D, E)** WT cells were cultured in the synthetic minimal YNB medium initially containing 2% glucose and one of the following supplements: 0.5% PE4, 100 µM S, or a mixture of 0.1% PE4 and 100 µM S. In the cultures supplemented with PE4 and/or S, ethanol was used as a vehicle at the final concentration of 2.5%. In the same experiment, WT cells were also subjected to ethanol-mock treatment by being cultured in the synthetic minimal YNB medium initially containing 2% glucose and 2.5% ethanol. Survival curves (D) and the mean and maximum lifespans (E) of chronologically aging WT cells cultured without a PE and S (cells were subjected to ethanol-mock treatment), with 0.5% PE4, with 100 µM S, or with the mixture of 0.1% PE4 and 100 µM S are shown. Data in D and E are presented as means ± SEM (n = 3; *p < 0.05; **p < 0.01; *** < 0.001). The CI values in E were calculated as follows: CI = CLS_{PE4}/CLS_{PE4+S} for both the mean and maximum CLS; the significance of a synergistic effect (i.e. CI < 1) is provided as the *p* value of the two-tailed *t* test for comparing the effect of a PE combination (i.e. CLS_{PE3+S}) to that of the HSA (i.e. CLS_{PE4} for both the mean and maximum CLS). Data for mock-treated WT cells are replicated in graphs D and E of Figures 1-14, Supplementary Figures 2-8 and Supplementary Figures 10-14. Data for WT cells cultured with 0.5% PE4 are replicated in graphs D and E of Figures 1-4, Figure 11 and Supplementary Figure 2. Data for WT cells cultured with 100 µM S are replicated in graphs D and E of Figure 9, Figure 10 and Supplementary Figures 10-12. **(F)** *p* Values for different pairs of survival curves of WT cells cultured in the presence of 0.5% PE4, 100 µM S, a mixture of 0.1% PE4 and 100 µM S, or in the absence of a PE and S (cells were subjected to ethanol-mock treatment) are shown. Survival curves shown in (D) were compared. Two survival curves were considered statistically different if the *p* value was less than 0.05. The *p* values for comparing pairs of survival curves using the logrank test were calculated as described in Materials and Methods. The *p* values displayed on a yellow color background indicate that 0.5% PE4, 100 µM S, and the mixture of 0.1% PE4 and 100 µM S significantly extend the CLS of WT cells. The *p* values displayed on a blue color background indicate that the CLS-extending efficiency of the mixture of 0.1% PE4 and 100 µM S significantly exceeds that of 0.5% PE4 or 100 µM S. Abbreviations: as in the legend to Figure 1.

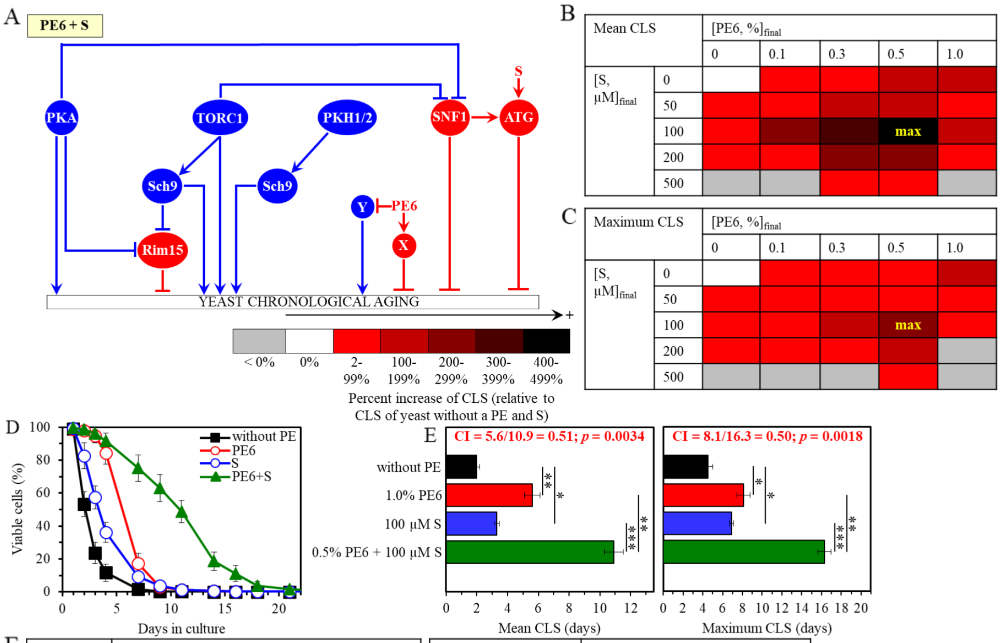

F

	p Values for pairs of survival curves		
	1.0% PE6	100 µM S	0.5% PE6 + 100 µM S
without PE	0.0048	0.0144	0.0003

	p Values for pairs of survival curves	
	1.0% PE6	100 µM S
0.5% PE6 + 100 µM S	0.0023	0.0009

Supplementary Figure 10: The longevity-extending efficiency of a mixture of 0.5% PE6 and 100 µM spermidine (S) statistically significantly exceeds those of PE6 and S, which were used at the optimal concentration of 1.0% or 100 µM (respectively). Thus, PE6 and S enhance the longevity-extending efficiency of each other. Hence, according to the highest single agent (HSA) model, PE6 and S act in synergy to extend longevity of chronologically aging yeast. (A) PE6 and S are known to regulate different nodes of the signaling network that controls the rate of yeast chronological aging. S stimulates the anti-aging ATG pathway, whereas PE6 modulates a presently unknown pro-aging or anti-aging node that may be integrated into this network. **(B, C)** WT cells were grown in the synthetic minimal YNB medium initially containing 2% glucose, with PE6 (at the final concentration of 0.1%, 0.3%, 0.5% or 1.0%) and/or S (at the final concentration of 50 µM, 100 µM, 200 µM or 500 µM), or without a PE and S. Effects of different concentrations of PE6 and S (added alone or in pairwise combinations) on the mean (B) or maximum (C) CLS of WT cells are shown. The table cell at the intersection of the column for 0.5% PE6 and the row for 100 µM S is marked "max" because the mixture of 0.5% PE6 and 100 µM S exhibits the highest extending effect on the mean and maximum lifespans of chronologically aging WT cells. **(D, E)** WT cells were cultured in the synthetic minimal YNB medium initially containing 2% glucose and one of the following supplements: 1.0% PE6, 100 µM S, or a mixture of 0.5% PE6 and 100 µM S. In the cultures supplemented with PE6 and/or S, ethanol was used as a vehicle at the final concentration of 2.5%. In the same experiment, WT cells were also subjected to ethanol-mock treatment by being cultured in the synthetic minimal YNB medium initially containing 2% glucose and 2.5% ethanol. Survival curves (D) and the mean and maximum lifespans (E) of chronologically aging WT cells cultured without a PE and S (cells were subjected to ethanol-mock treatment), with 1.0% PE6, with 100 µM S, or with the mixture of 0.5% PE6 and 100 µM S are shown. Data in D and E are presented as means ± SEM (n = 3; *p < 0.05; **p < 0.01; *** < 0.001). The CI values in E were calculated as follows: CI = CLS_{PE6}/CLS_{PE6+S} for both the mean and maximum CLS; the significance of a synergistic effect (i.e. CI < 1) is provided as the *p* value of the two-tailed *t* test for comparing the effect of a PE combination (i.e. CLS_{PE6+S}) to that of the HSA (i.e. CLS_{PE6} for both the mean and maximum CLS). Data for mock-treated WT cells are replicated in graphs D and E of Figures 1-14, Supplementary Figures 2-9 and Supplementary Figures 11-14. Data for WT cells cultured with 1.0% PE6 are replicated in graphs D and E of Figure 7, Figure 12, Supplementary Figure 2, Supplementary Figure 3, Supplementary Figure 5 and Supplementary Figure 6. Data for WT cells cultured with 100 µM S are replicated in graphs D and E of Figure 9, Figure 10, Supplementary Figure 9, Supplementary Figure 11 and Supplementary Figure 12. **(F)** *p* Values for different pairs of survival curves of WT cells cultured in the presence of 1.0% PE6, 100 µM S, a mixture of 0.5% PE6 and 100 µM S, or in the absence of a PE and S (cells were subjected to ethanol-mock treatment) are shown. Survival curves shown in (D) were compared. Two survival curves were considered statistically different if the *p* value was less than 0.05. The *p* values for comparing pairs of survival curves using the logrank test were calculated as described in Materials and Methods. The *p* values displayed on a yellow color background indicate that 1.0% PE6, 100 µM S, and the mixture of 0.5% PE6 and 100 µM S significantly extend the CLS of WT cells. The *p* values displayed on a blue color background indicate that the CLS-extending efficiency of the mixture of 0.5% PE6 and 100 µM S significantly exceeds that of 1.0% PE6 or 100 µM S. Abbreviations: as in the legend to Figure 1.

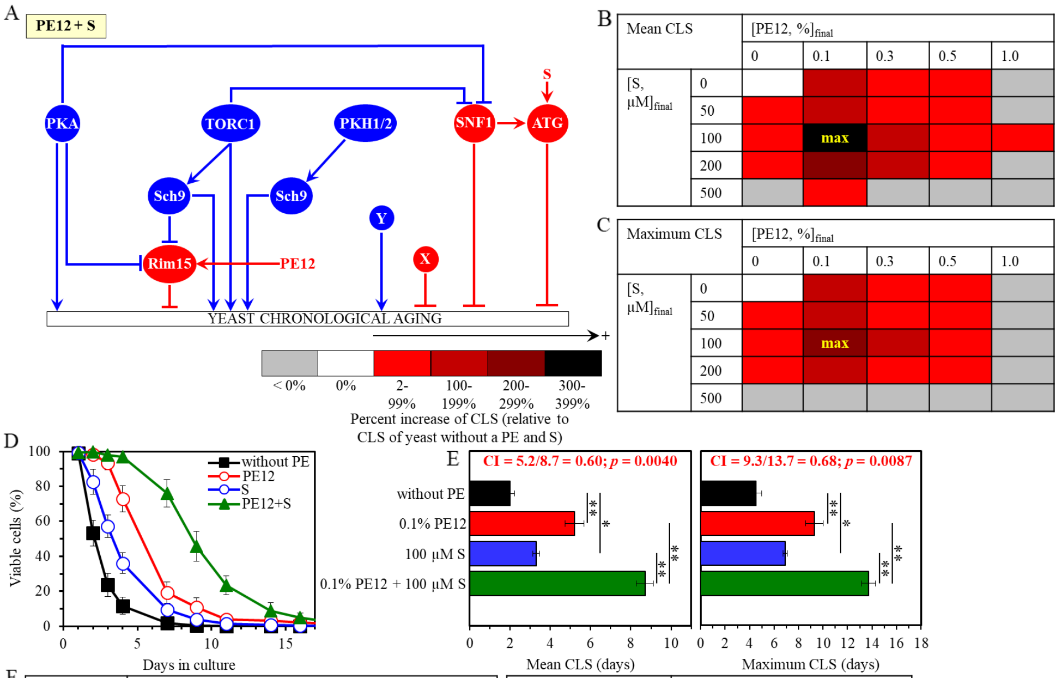

F

	p Values for pairs of survival curves		
	0.1% PE12	100 µM S	0.1% PE12 + 100 µM S
without PE	0.0041	0.0144	0.0003

	p Values for pairs of survival curves	
	0.1% PE12	100 µM S
0.1% PE12 + 100 µM S	0.0054	0.0012

Supplementary Figure 11: The longevity-extending efficiency of a mixture of 0.1% PE12 and 100 µM spermidine (S) statistically significantly exceeds those of PE12 and S, which were used at the optimal concentration of 0.1% or 100 µM (respectively). Thus, PE12 and S enhance the longevity-extending efficiency of each other. Hence, according to the highest single agent (HSA) model, PE12 and S act in synergy to extend longevity of chronologically aging yeast. **(A)** PE12 and S are known to regulate different nodes of the signaling network that controls the rate of yeast chronological aging. PE12 activates the anti-aging protein kinase Rim15, whereas S stimulates the anti-aging ATG pathway. **(B, C)** WT cells were grown in the synthetic minimal YNB medium initially containing 2% glucose, with PE12 (at the final concentration of 0.1%, 0.3%, 0.5% or 1.0%) and/or S (at the final concentration of 50 µM, 100 µM, 200 µM or 500 µM), or without a PE and S. Effects of different concentrations of PE12 and S (added alone or in pairwise combinations) on the mean (B) or maximum (C) CLS of WT cells are shown. The table cell at the intersection of the column for 0.1% PE12 and the row for 100 µM S is marked "max" because the mixture of 0.1% PE12 and 100 µM S exhibits the highest extending effect on the mean and maximum lifespans of chronologically aging WT cells. **(D, E)** WT cells were cultured in the synthetic minimal YNB medium initially containing 2% glucose and one of the following supplements: 0.1% PE12, 100 µM S, or a mixture of 0.1% PE12 and 100 µM S. In the cultures supplemented with PE12 and/or S, ethanol was used as a vehicle at the final concentration of 2.5%. In the same experiment, WT cells were also subjected to ethanol-mock treatment by being cultured in the synthetic minimal YNB medium initially containing 2% glucose and 2.5% ethanol. Survival curves (D) and the mean and maximum lifespans (E) of chronologically aging WT cells cultured without a PE and S (cells were subjected to ethanol-mock treatment), with 0.1% PE12, with 100 µM S, or with the mixture of 0.1% PE12 and 100 µM S are shown. Data in D and E are presented as means ± SEM (n = 3; *p < 0.05; **p < 0.01). The CI values in E were calculated as follows: $CI = CLS_{PE12}/CLS_{PE12+S}$ for both the mean and maximum CLS; the significance of a synergistic effect (i.e. CI < 1) is provided as the *p* value of the two-tailed *t* test for comparing the effect of a PE combination (i.e. CLS_{PE12+S}) to that of the HSA (i.e. CLS_{PE12} for both the mean and maximum CLS). Data for mock-treated WT cells are replicated in graphs D and E of Figures 1-14, Supplementary Figures 2-10 and Supplementary Figures 12-14. Data for WT cells cultured with 0.1% PE12 are replicated in graphs D and E of Figure 3, Figure 7, Figure 8, Figure 13, Supplementary Figure 4, Supplementary Figure 8 and Supplementary Figure 12. Data for WT cells cultured with 100 µM S are replicated in graphs D and E of Figure 9, Figure 10, Supplementary Figure 9, Supplementary Figure 10 and Supplementary Figure 12. **(F)** *p* Values for different pairs of survival curves of WT cells cultured in the presence of 0.1% PE12, 100 µM S, a mixture of 0.1% PE12 and 100 µM S, or in the absence of a PE and S (cells were subjected to ethanol-mock treatment) are shown. Survival curves shown in (D) were compared. Two survival curves were considered statistically different if the *p* value was less than 0.05. The *p* values for comparing pairs of survival curves using the logrank test were calculated as described in Materials and Methods. The *p* values displayed on a yellow color background indicate that 0.1% PE12, 100 µM S, and the mixture of 0.1% PE12 and 100 µM S significantly extend the CLS of WT cells. The *p* values displayed on a blue color background indicate that the CLS-extending efficiency of the mixture of 0.1% PE12 and 100 µM S significantly exceeds that of 0.1% PE12 or 100 µM S. Abbreviations: as in the legend to Figure 1.

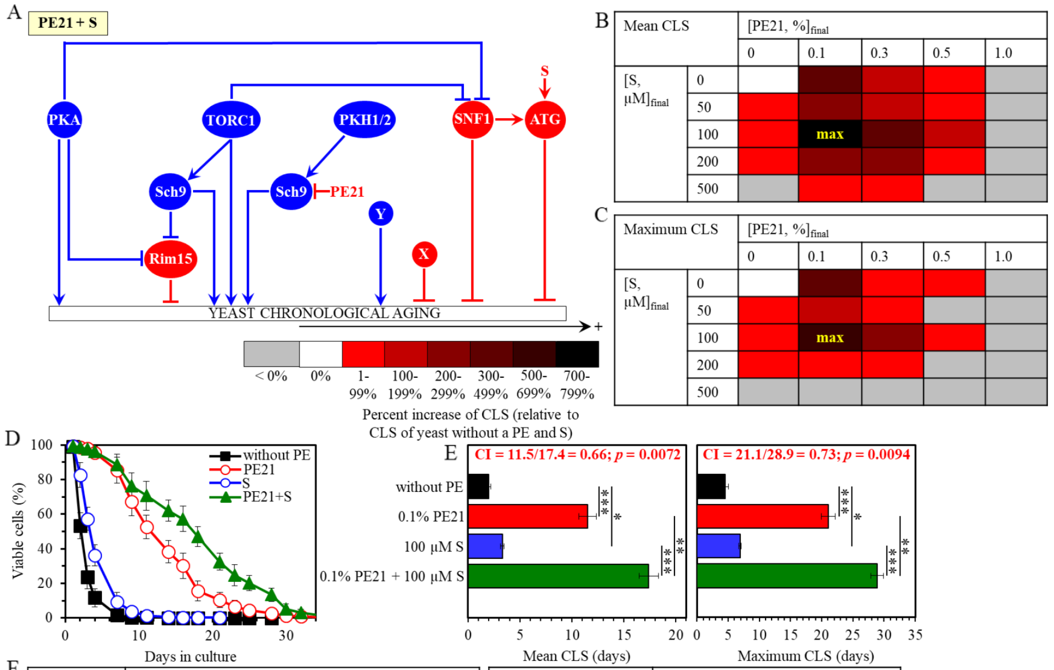

F

	p Values for pairs of survival curves		
	0.1% PE21	100 µM S	0.1% PE21 + 100 µM S
without PE	**0.0008**	**0.0144**	**0.0002**

	p Values for pairs of survival curves	
	0.1% PE21	100 µM S
0.1% PE21 + 100 µM S	**0.0047**	**0.0009**

Supplementary Figure 12: The longevity-extending efficiency of a mixture of 0.1% PE21 and 100 µM spermidine (S) statistically significantly exceeds those of PE21 and S, which were used at the optimal concentration of 0.1% or 100 µM (respectively). Thus, PE21 and S enhance the longevity-extending efficiency of each other. Hence, according to the highest single agent (HSA) model, PE21 and S act in synergy to extend longevity of chronologically aging yeast. (A) PE21 and S are known to regulate different nodes of the signaling network that controls the rate of yeast chronological aging. PE21 mitigates a form of the pro-aging protein kinase Sch9 that is activated by the pro-aging PKH1/2 pathway, whereas S stimulates the anti-aging ATG pathway. **(B, C)** WT cells were grown in the synthetic minimal YNB medium initially containing 2% glucose, with PE21 (at the final concentration of 0.1%, 0.3%, 0.5% or 1.0%) and/or S (at the final concentration of 50 µM, 100 µM, 200 µM or 500 µM), or without a PE and S. Effects of different concentrations of PE21 and S (added alone or in pairwise combinations) on the mean (B) or maximum (C) CLS of WT cells are shown. The table cell at the intersection of the column for 0.1% PE21 and the row for 100 µM S is marked "max" because the mixture of 0.1% PE21 and 100 µM S exhibits the highest extending effect on the mean and maximum lifespans of chronologically aging WT cells. **(D, E)** WT cells were cultured in the synthetic minimal YNB medium initially containing 2% glucose and one of the following supplements: 0.1% PE21, 100 µM S, or a mixture of 0.1% PE21 and 100 µM S. In the cultures supplemented with PE21 and/or S, ethanol was used as a vehicle at the final concentration of 2.5%. In the same experiment, WT cells were also subjected to ethanol-mock treatment by being cultured in the synthetic minimal YNB medium initially containing 2% glucose and 2.5% ethanol. Survival curves (D) and the mean and maximum lifespans (E) of chronologically aging WT cells cultured without a PE and S (cells were subjected to ethanol-mock treatment), with 0.1% PE21, with 100 µM S, or with the mixture of 0.1% PE21 and 100 µM S are shown. Data in D and E are presented as means ± SEM (n = 3; *p < 0.05; **p < 0.01; ***p < 0.001). The CI values in E were calculated as follows: $CI = CLS_{PE21}/CLS_{PE21+S}$ for both the mean and maximum CLS; the significance of a synergistic effect (i.e. CI < 1) is provided as the *p* value of the two-tailed *t* test for comparing the effect of a PE combination (i.e. CLS_{PE21+S}) to that of the HSA (i.e. CLS_{PE21} for both the mean and maximum CLS). Data for mock-treated WT cells are replicated in graphs D and E of Figures 1-14, Supplementary Figures 2-11, Supplementary Figure 13 and Supplementary Figure 14. Data for WT cells cultured with 0.1% PE21 are replicated in graphs D and E of Figure 4, Figure 6, Figure 14 and Supplementary Figures 6-8. Data for WT cells cultured with 100 µM S are replicated in graphs D and E of Figure 9, Figure 10 and Supplementary Figures 9-11. **(F)** *p* Values for different pairs of survival curves of WT cells cultured in the presence of 0.1% PE21, 100 µM S, a mixture of 0.1% PE21 and 100 µM S, or in the absence of a PE and S (cells were subjected to ethanol-mock treatment) are shown. Survival curves shown in (D) were compared. Two survival curves were considered statistically different if the *p* value was less than 0.05. The *p* values for comparing pairs of survival curves using the logrank test were calculated as described in Materials and Methods. The *p* values displayed on a yellow color background indicate that 0.1% PE21, 100 µM S, and the mixture of 0.1% PE21 and 100 µM S significantly extend the CLS of WT cells. The *p* values displayed on a blue color background indicate that the CLS-extending efficiency of the mixture of 0.1% PE21 and 100 µM S significantly exceeds that of 0.1% PE21 or 100 µM S. Abbreviations: as in the legend to Figure 1.

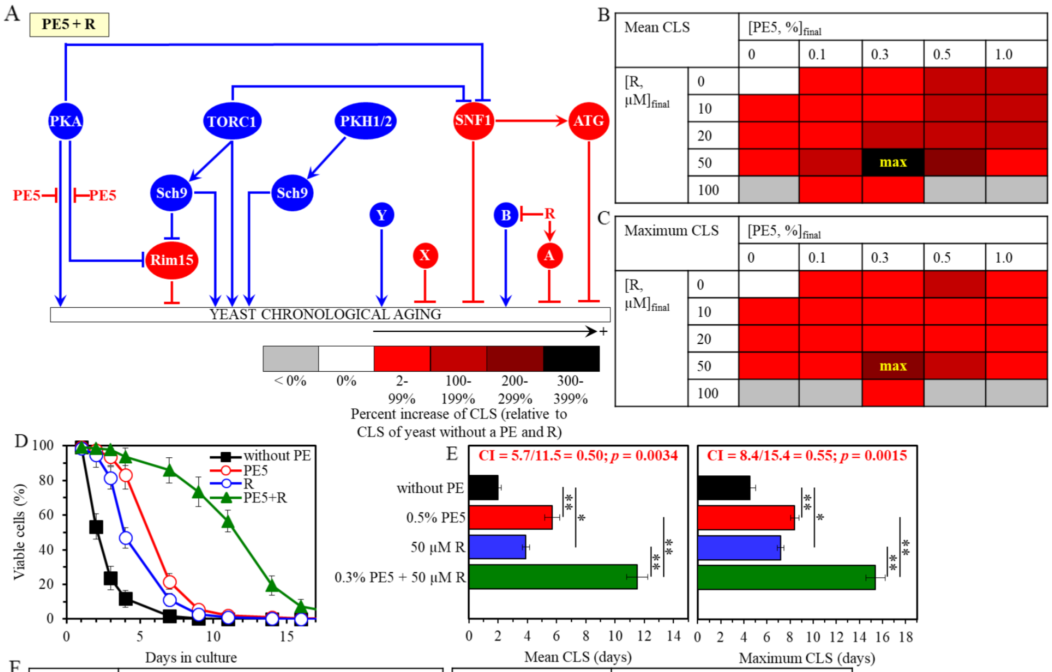

F

	p Values for pairs of survival curves		
	0.5% PE5	50 µM R	0.3% PE5 + 50 µM R
without PE	0.0039	0.00112	0.0003

	p Values for pairs of survival curves	
	0.5% PE5	50 µM R
0.3% PE5 + 50 µM R	0.0023	0.0011

Supplementary Figure 13: The longevity-extending efficiency of a mixture of 0.3% PE5 and 50 µM resveratrol (R) statistically significantly exceeds those of PE5 and R, which were used at the optimal concentration of 0.5% or 50 µM (respectively). Thus, PE5 and R enhance the longevity-extending efficiency of each other. Hence, according to the highest single agent (HSA) model, PE5 and R act in synergy to extend longevity of chronologically aging yeast. **(A)** PE5 and R are known to regulate different nodes of the signaling network that controls the rate of yeast chronological aging. PE5 mitigates two different branches of the pro-aging PKA pathway, whereas R modulates a presently unknown pro-aging or anti-aging node that may be integrated into this signaling network. **(B, C)** WT cells were grown in the synthetic minimal YNB medium initially containing 2% glucose, with PE5 (at the final concentration of 0.1%, 0.3%, 0.5% or 1.0%) and/or R (at the final concentration of 10 µM, 20 µM, 50 µM or 100 µM), or without a PE and R. Effects of different concentrations of PE5 and R (added alone or in pairwise combinations) on the mean (B) or maximum (C) CLS of WT cells are shown. The table cell at the intersection of the column for 0.3% PE5 and the row for 50 µM R is marked "max" because the mixture of 0.3% PE5 and 50 µM R exhibits the highest extending effect on the mean and maximum lifespans of chronologically aging WT cells. **(D, E)** WT cells were cultured in the synthetic minimal YNB medium initially containing 2% glucose and one of the following supplements: 0.5% PE5, 50 µM R, or a mixture of 0.3% PE5 and 50 µM R. In the cultures supplemented with PE5 and/or R, ethanol was used as a vehicle at the final concentration of 2.5%. In the same experiment, WT cells were also subjected to ethanol-mock treatment by being cultured in the synthetic minimal YNB medium initially containing 2% glucose and 2.5% ethanol. Survival curves (D) and the mean and maximum lifespans (E) of chronologically aging WT cells cultured without a PE and R (cells were subjected to ethanol-mock treatment), with 0.5% PE5, with 50 µM R, or with the mixture of 0.3% PE5 and 50 µM R are shown. Data in D and E are presented as means ± SEM (n = 3; *p < 0.05; **p < 0.01). The CI values in E were calculated as follows: CI = CLS_{PE5}/CLS_{PE5+R} for both the mean and maximum CLS; the significance of a synergistic effect (i.e. CI < 1) is provided as the *p* value of the two-tailed *t* test for comparing the effect of a PE combination (i.e. CLS_{PE5+R}) to that of the HSA (i.e. CLS_{PE5} for both the mean and maximum CLS). Data for mock-treated WT cells are replicated in graphs D and E of Figures 1-14, Supplementary Figures 2-12 and Supplementary Figure 14. Data for WT cells cultured with 0.5% PE5 are replicated in graphs D and E of Figure 1, Figure 5, Figure 6, Figure 9, Supplementary Figure 3, Supplementary Figure 4 and Supplementary Figure 13. Data for WT cells cultured with 50 µM R are replicated in graphs D and E of Figures 11-14 and Supplementary Figure 14. **(F)** *p* Values for different pairs of survival curves of WT cells cultured in the presence of 0.5% PE5, 50 µM R, a mixture of 0.3% PE5 and 50 µM R, or in the absence of a PE and R (cells were subjected to ethanol-mock treatment) are shown. Survival curves shown in (D) were compared. Two survival curves were considered statistically different if the *p* value was less than 0.05. The *p* values for comparing pairs of survival curves using the logrank test were calculated as described in Materials and Methods. The *p* values displayed on a yellow color background indicate that 0.5% PE5, 50 µM R, and the mixture of 0.3% PE5 and 50 µM R significantly extend the CLS of WT cells. The *p* values displayed on a blue color background indicate that the CLS-extending efficiency of the mixture of 0.3% PE5 and 50 µM R significantly exceeds that of 0.5% PE5 or 50 µM R. Abbreviations: as in the legend to Figure 1.

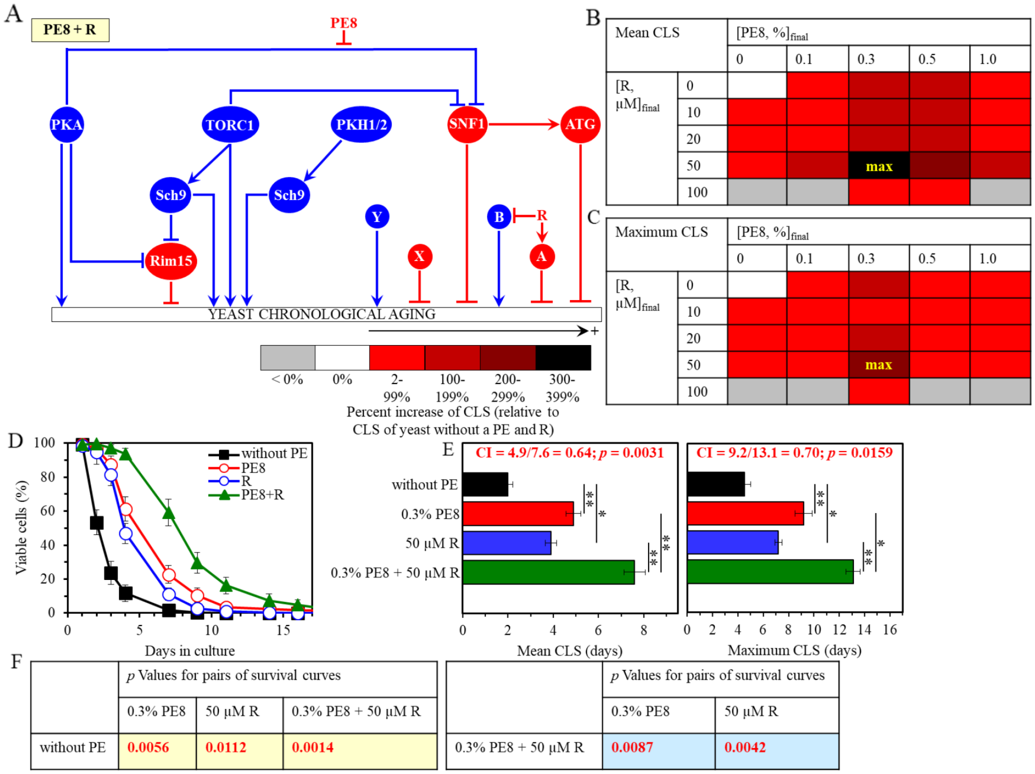

F

	p Values for pairs of survival curves		
	0.3% PE8	50 µM R	0.3% PE8 + 50 µM R
without PE	0.0056	0.0112	0.0014

	p Values for pairs of survival curves	
	0.3% PE8	50 µM R
0.3% PE8 + 50 µM R	0.0087	0.0042

Supplementary Figure 14: The longevity-extending efficiency of a mixture of 0.3% PE8 and 50 µM resveratrol (R) statistically significantly exceeds those of PE8 and R, which were used at the optimal concentration of 0.3% or 50 µM (respectively). Thus, PE8 and R enhance the longevity-extending efficiency of each other. Hence, according to the highest single agent (HSA) model, PE8 and R act in synergy to extend longevity of chronologically aging yeast. (A) PE8 and R are known to regulate different nodes of the signaling network that controls the rate of yeast chronological aging. PE8 weakens the restraining action of the pro-aging PKA pathway on the anti-aging SNF1 pathway, whereas R modulates a presently unknown pro-aging or anti-aging node that may be integrated into this signaling network. **(B, C)** WT cells were grown in the synthetic minimal YNB medium initially containing 2% glucose, with PE8 (at the final concentration of 0.1%, 0.3%, 0.5% or 1.0%) and/or R (at the final concentration of 10 µM, 20 µM, 50 µM or 100 µM), or without a PE and R. Effects of different concentrations of PE8 and R (added alone or in pairwise combinations) on the mean (B) or maximum (C) CLS of WT cells are shown. The table cell at the intersection of the column for 0.3% PE8 and the row for 50 µM R is marked "max" because the mixture of 0.3% PE8 and 50 µM R exhibits the highest extending effect on the mean and maximum lifespans of chronologically aging WT cells. **(D, E)** WT cells were cultured in the synthetic minimal YNB medium initially containing 2% glucose and one of the following supplements: 0.3% PE8, 50 µM R, or a mixture of 0.3% PE8 and 50 µM R. In the cultures supplemented with PE8 and/or R, ethanol was used as a vehicle at the final concentration of 2.5%. In the same experiment, WT cells were also subjected to ethanol-mock treatment by being cultured in the synthetic minimal YNB medium initially containing 2% glucose and 2.5% ethanol. Survival curves (D) and the mean and maximum lifespans (E) of chronologically aging WT cells cultured without a PE and R (cells were subjected to ethanol-mock treatment), with 0.3% PE8, with 50 µM R, or with the mixture of 0.3% PE8 and 50 µM R are shown. Data in D and E are presented as means ± SEM (n = 3; *p < 0.05; **p < 0.01). The CI values in E were calculated as follows: CI = CLS_{PE8}/CLS_{PE8+R} for both the mean and maximum CLS; the significance of a synergistic effect (i.e. CI < 1) is provided as the *p* value of the two-tailed *t* test for comparing the effect of a PE combination (i.e. CLS_{PE8+R}) to that of the HSA (i.e. CLS_{PE8} for both the mean and maximum CLS). Data for mock-treated WT cells are replicated in graphs D and E of Figures 1-14 and Supplementary Figures 2-13. Data for WT cells cultured with 0.3% PE8 are replicated in graphs D and E of Figure 2, Figure 5, Figure 8, Figure 10, Supplementary Figure 5 and Supplementary Figure 7. Data for WT cells cultured with 50 µM R are replicated in graphs D and E of Figures 11-14 and Supplementary Figure 13. **(F)** *p* Values for different pairs of survival curves of WT cells cultured in the presence of 0.3% PE8, 50 µM R, a mixture of 0.3% PE8 and 50 µM R, or in the absence of a PE and R (cells were subjected to ethanol-mock treatment) are shown. Survival curves shown in (D) were compared. Two survival curves were considered statistically different if the *p* value was less than 0.05. The *p* values for comparing pairs of survival curves using the logrank test were calculated as described in Materials and Methods. The *p* values displayed on a yellow color background indicate that 0.3% PE8, 50 µM R, and the mixture of 0.3% PE8 and 50 µM R significantly extend the CLS of WT cells. The *p* values displayed on a blue color background indicate that the CLS-extending efficiency of the mixture of 0.3% PE8 and 50 µM R significantly exceeds that of 0.3% PE8 or 50 µM R. Abbreviations: as in the legend to Figure 1.
